# Blood-based biomarkers and delirium in critically ill adults in the ICU: a systematic review

**DOI:** 10.1186/s40635-026-00894-5

**Published:** 2026-04-20

**Authors:** Julie Kathrine Ryberg Sankel, Naia Bech Cranø, Pär Ingemar Johansson, Lars Peter Kloster Andersen

**Affiliations:** 1 Centre for Anaesthesiological Research, Department of Anaesthesiology, Zealand University Hospital Køge, Lykkebækvej 1, 4600 Køge, Denmark; 2 Center for Endotheliomics, CAG, Department of Clinical Immunology, Copenhagen University Hospital-Rigshospitalet, Copenhagen, Denmark; 3 Department of Clinical Medicine, University of Copenhagen, Copenhagen, Denmark

**Keywords:** Delirium, Blood biomarkers, Intensive care unit, ICU-acquired delirium, Systematic review

## Abstract

**Purpose:**

Delirium is frequent and associated with increased health expenditures, unfavourable outcomes and increased mortality. Diagnosis currently relies on clinical scales and is often missed. However, blood-based biomarkers may represent a feasible tool to enhance risk stratification, early diagnosis, and monitoring. We aimed to identify blood-based biomarkers associated with delirium in adult intensive care unit patients, to provide a structured summary of the evidence, and to highlight gaps of knowledge to inform future research.

**Methods:**

A systematic literature search was performed in PubMed, EMBASE, The Cochrane Library*,* and Web of Science from inception to 3rd September 2025 with no date or language restrictions. Eligible studies were observational, assessing associations between blood-based biomarkers and delirium in critically ill adults admitted to an intensive care unit. The Newcastle–Ottawa Scale was applied to assess study quality. Data were qualitatively synthesized in a systematic narrative synthesis.

**Results:**

Out of 2130 records including duplicates, 61 studies were included, comprising 39,355 adult patients, of whom 16,762 were screened positive for delirium. Delirium definition, assessment tools and frequency, reported delirium outcomes, and timing of biomarker sampling varied. In total, 153 blood-based biomarkers were identified, 101 were significantly associated with delirium and 40 were associated with delirium across two studies or more. Study quality was generally poor and substantial study heterogeneity limited comparability.

**Conclusion:**

Current evidence does not support clinical use of any biomarker. Poor study quality, small sample sizes, and considerable methodological heterogeneity limit interpretation, generalizability, validation, and firm conclusions. Standardized methodology and international consensus are essential in future research.

**Supplementary Information:**

The online version contains supplementary material available at 10.1186/s40635-026-00894-5.

## Introduction

Delirium is a neuropsychiatric syndrome with an incidence of up to 87% in patients admitted to the intensive care unit (ICU) [1]. It is characterized by fluctuating disturbances in attention and awareness with acute or subacute onset [2]. Clinically, delirium presents as a cluster of cognitive and behavioural symptoms, varying substantially in both severity and manifestation [1]. ICU-acquired delirium influences patient trajectory and is associated with serious adverse outcomes such as increased complication rates [3, 4], markedly increased health care costs [5] and increased mortality [3, 4].

The pathophysiology of delirium remains poorly understood and is thought to involve complex interactions of multiple mechanisms, none of which has been established as causal [1, 6]. Disruption of neuronal activity likely occurs through a final common pathway involving impaired oxidative metabolism [6], aberrant stress response [6], circadian dysregulation [6], anatomic deficits [7], neurotransmitter imbalances [7], metabolic derangements [8], and neuroinflammation [6]. The risk of developing delirium is influenced by a complex interplay between predisposing and precipitating factors [9]. ICU patients are particularly vulnerable due to the cumulative burden of risk factors related to severity of their illness and the ICU treatment course [10, 11].

Despite its clinical significance, the heterogeneous aetiology of delirium poses substantial challenges for diagnosis, effective prophylaxis, and therapeutic management. Current guidelines [12] recommend daily delirium assessment employing validated assessment scales [13, 14]. However, symptom variability and fluctuating severity may impede recognition [15], and furthermore, these scales are inapplicable in patients with severely altered consciousness [16].

Biomarkers are objectively measured biological indicators of a physiological or pathological process or a response to a therapeutic intervention [17]. They may improve understanding of the pathophysiology, and potentially enhance risk stratification, monitoring of disease progression, evaluation of therapeutic interventions, and ultimately improve patient outcomes [17–19]. Blood-based biomarkers appear particularly advantageous due to accessibility and suitability for repeated sampling in critically ill patients. Nevertheless, the intricate, dynamic nature of delirium, along with heterogeneous study designs, has hampered biomarker identification [20]. While several systematic reviews on delirium biomarkers have been performed recently [20–24], very limited knowledge exists concerning the ICU patient population, despite indications that biomarker profiles may differ between critically ill and non-critically ill patients [25].

This systematic review summarizes the current evidence on blood-based biomarkers associated with delirium in adult ICU patients. We aim to provide a comprehensive overview of the literature, identify gaps in existing knowledge, and suggest directions for future research.

## Materials and methods

This systematic review adheres to the ‘Preferred Reporting Items for Systematic Reviews and Meta-Analyses’ (PRISMA) 2020 guideline [26]. If deviations from the guideline occurred, these are outlined in the text. The review protocol was registered in PROSPERO (CRD420251166045).

### Eligibility criteria

The eligibility criteria were defined according to a modified ‘Population, Intervention, Comparator, Outcome’ (PICO) framework [27]. Observational studies assessing blood-based biomarkers and their association with delirium in critically ill adults (> 18 years) during ICU admission were included in the review. Delirium had to be assessed with a clinical screening tool. Studies were included if they compared blood-based biomarker levels in patients with and without delirium *OR* assessed biomarker levels in relation to delirium trajectory in the absence of a comparator group. There were no restrictions with regard to publication year. Studies written in a language other than English or without an available full-text article were excluded. Randomized controlled trials, case reports and abstracts were excluded. Studies investigating delirium tremens and studies in patients with planned ICU admission were also excluded.

### Information sources

A systematic literature search was performed in the following databases: PubMed *(National Library of Medicine, 1946–present),* EMBASE *(Ovid, 1974–present),* The Cochrane Library *(Cochrane, 1992–present)* and Web of Science *(Clarivate, 1900–present).* Moreover, the reference lists of included studies and relevant reviews were manually assessed for potentially relevant literature.

### Search strategy

The search strategy was developed for PubMed by combining text-words and ‘Medical Subject Headings’ (MeSH) terms based on a PICO approach employing a block search strategy. An academic information specialist reviewed the search strategy. Boolean operators were employed to combine search terms such as “delirium”, “critical illness”, “intensive care unit”, “biomarkers” *(detailed search protocol available in Appendix A in Supplementary Materials).* No date or language limitations were applied. The search strategy was subsequently adapted with appropriate amendments for the other databases.

### Selection process

Relevant retrieved literature was uploaded to Covidence *(Veritas Health Innovation Ltd)* [28] for automated removal of duplicates. Two independent reviewers (JKRS, NCB) screened titles and abstracts, followed by full-text assessment of the studies adhering to the eligibility criteria. Reasons for final exclusion were documented. In cases of uncertainty, the senior author (LPKA) was consulted for a final decision. Reference lists of the included studies were screened, and potentially relevant titles were selected for review of abstracts and subsequently full-text evaluation if they fulfilled the eligibility criteria.

### Data extraction

One review author (JKRS) independently extracted the data from included studies. The senior author (LPKA) was consulted regarding data interpretation in case of uncertainty and also checked the final data extracted before the beginning of the evidence synthesis.

The following variables were extracted: (1) study characteristics (author, year of publication, study design, ICU setting, eligibility criteria); (2) patient characteristics (total number of patients, gender, number of patients with delirium); (3) delirium assessment (assessment tool, frequency of delirium assessment, definition of delirium); (4) biomarker sampling (timing and sampling site); (5) delirium outcomes; (6) additional patient characteristics and outcomes (admission diagnosis, comorbidities, disease severity scores, ICU treatments and medications, ICU- and hospital length of stay, recovery outcomes, mortality), (7) delirium biomarkers (biomarkers investigated with statistical association analyses in relation to delirium), and (8) main study conclusions.

Results of multivariate analyses were preferred for data extraction. In case multivariate analysis was not performed, results of univariate analyses were extracted. In cases of missing data, these were reported as ‘not specified’, as no study investigators were contacted for additional information.

### Study risk of bias assessment

Study quality was assessed by two independent reviewers (JKRS, NCB) employing the Newcastle–Ottawa Scale (NOS) [29]. NOS evaluates the methodological quality of studies based on three levels: (1) selection of study groups, (2) comparability, and (3) ascertainment of either exposure or outcome of interest. The scale ranges from 0 to 9 and a star is assigned when ‘high’ quality choices are identified. The scores for each study were then converted and categorized as low-, fair- or high quality according to the Agency for Health Research and Quality (AHRQ) standards [30], following conventions commonly applied in systematic reviews [31–33].

Prior to application, we adapted the criteria for awarding a star in each domain to obtain the best fit to the purpose of this review. A dropout rate below 20% was considered acceptable, and daily delirium assessments conducted throughout the entire ICU stay were deemed indicative of a sufficient follow-up period. Age, disease severity, and neurological status were specified as the most important confounders. Any disagreements between the two reviewers (JKRS, NCB) were resolved through discussion to reach consensus. If consensus could not be reached or if any uncertainties arose, the senior author (LPKA) was consulted to facilitate final agreement.

### Synthesis methods

Collected data were synthesized qualitatively in a systematic narrative synthesis. Given the large number of serum biomarkers evaluated, they were thematically categorized as follows: haematology, coagulation and endothelial dysfunction, inflammatory, infectious and immunological markers, neurobiological markers, organ injury, metabolic and endocrine function, amino acid metabolism, and omics-based exploratory markers. Appendix B (see Supplementary Materials 1) provides a list of the assessed biomarkers (with abbreviations) in each category. Studies and data were included and reported in tables regardless of the evaluated study quality. No meta-analysis was performed due to considerable clinical and methodological heterogeneity of the included studies [34, 35].

### Certainty of evidence assessment

Assessment of the overall certainty of the gathered evidence, e.g. applying the “Grading of Recommendations, Assessment, Development and Evaluations” (GRADE) [36], was not performed.

## Results

### Search results

**Fig. 1:**
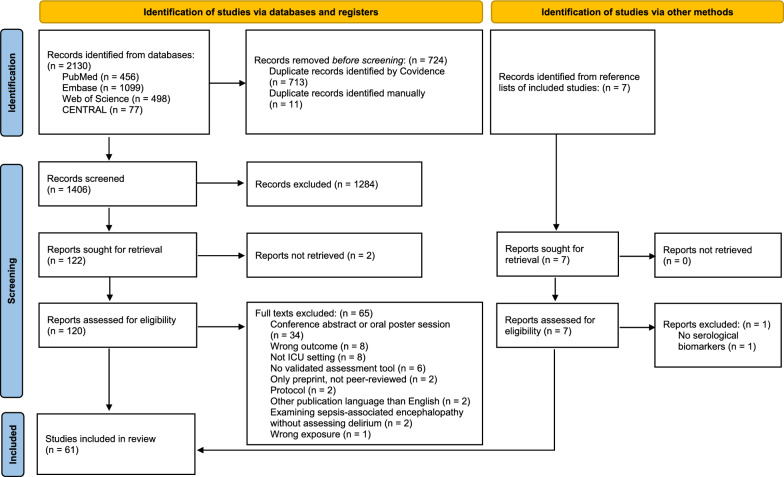
PRISMA 2020 flow diagram for new systematic reviews which included searches of databases, registers and other sources. Source: Page et al. [26]. This work is licensed under CC BY 4.0. To view a copy of this license, visit https://creativecommons.org/licenses/by/4.0/

A total of 2130 records were identified, including 713 duplicates detected by Covidence and 11 identified manually. After duplicate removal, 1406 records were screened based on title and abstract according to the eligibility criteria. Additionally, seven records were identified through manual search of the reference lists. Subsequently, 122 reports were sought for retrieval, of which 120 were available and assessed in full text. Sixty-five were excluded due to publication type (conference abstracts, protocols), publication in a language other than English, no validated assessment scale, only available as a preprint or due to irrelevant outcome, exposure, study population or setting. In total, 61 studies [37–97] were included for data extraction (see Fig. 1, PRISMA flowchart).

### Study characteristics

**Fig. 2:**
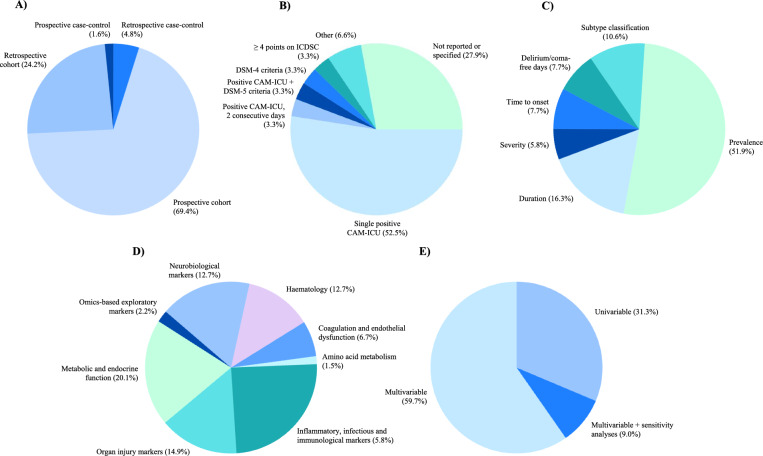
Summary figure. Figure illustrating key characteristics of the included studies. **A** Distribution of study designs among the included studies. **B** Distribution of delirium definitions. *“Others”* refers to delirium definitions based on medical records, including CAM-ICU combined with clinical or progress note review. Studies were categorized as ‘not specified’ if no explicit definition of delirium was provided. **C** Distribution of reported delirium outcomes. **D** Distribution of biomarker categories assessed across all biomarker measurements in the included studies. Individual studies frequently assessed biomarkers from more than one category and could therefore contribute to multiple categories. Percentages reflect the relative frequency of biomarker categories assessed. **E** Distribution of statistical analysis approaches.

The included observational studies comprised 57 cohort studies [37–56, 59–62, 64–82, 84–97] and four case–control studies [57, 58, 63, 83]. Forty-three studies (70%) applied a prospective study design [37, 38, 41–45, 48, 49, 51, 52, 54, 59, 61–82, 84–86, 91–95], 17 (28%) applied a retrospective study design [39, 40, 46, 47, 50, 53, 55, 57, 58, 60, 83, 87–90, 96, 97] and one study (2%) included both a prospective and a retrospective cohort [56]. Figure 2 illustrates the distribution of study designs. The studies were published from 2006 to 2025 and were conducted across five continents and in 19 different countries, most commonly in the USA (*n* = 18), China (*n* = 12) and Brazil (*n* = 5).

Sample sizes ranged from 16 to 7,812 participants. In total, 39,355 adult ICU patients were included, of whom 16,762 screened positive for delirium. Some studies included overlapping patient populations derived from the same parent cohort [42, 45, 59, 61, 69, 71, 78, 82, 84]. Thirty-one studies (51%) were set in mixed ICUs [42, 48, 50, 51, 53–55, 57, 59–62, 64, 66, 68–71, 73–75, 77, 78, 81–84, 93], 13 (21%) in medical ICUs [38, 43, 45–47, 49, 52, 72, 80, 92, 94, 96, 97], two (3%) in surgical ICUs [44, 58] and 15 studies (25%) did not specify their ICU setting [37, 39–41, 56, 63, 65, 67, 76, 79, 85–91, 95]. Further details on individual study characteristics are provided in Table 1.

**Table 1:** Study characteristics

Author	Year	Study design	Type of ICU	Inclusion criteria	Exclusion criteria	Number of patients	Gender (M/F) (%)	Patients with delirium (%)
Pham et al.	2025	Cohort (P)	Med	Acute respiratory failure requiring invasive mechanical ventilation; diagnosis of a primary pulmonary condition; expected need for ICU-level care for > 48 h;	Pregnancy; incarceration; immunocompromised patients; active use of immunosuppressive agents; pre-existing prescription for home oxygen; palliative care patients*; prisoners; admission haemoglobin of < 8 g/dL or transfusion requirement	100	76/24	73
Fayssoil et al.	2025	Cohort (R)	Med	> 75 years old; admitted to cardiac ICU	*NS*	451	47/53	18
Zhang et al.	2025	Cohort (R)	*NS*	≥ 18 years old; acute kidney injury; first ICU admission; ICU stay > 24 h	Psychiatric disorder; primary brain injury; disorders affecting consciousness; alcohol/drug abuse; missing laboratory data on glucose and triglycerides from ICU admission until onset of delirium	2919	66/34	47
Wang et al.	2025	Cohort (R)	*NS*	Ischemic stroke according to ICD-9 and -10	Dementia; history of schizophrenia; infections; trauma; allergies; neoplasms; missing laboratory data on NLR, PLR and LMR from first day of ICU admission	1436	52/48	15
Viegas et al.	2024	Cohort (P)	*NS*	PCR-confirmed COVID-19	History of psychiatric disorders; palliative care patients*; administration of benzodiazepines prior to admission; delirium upon or prior to the ICU admission	134	66/34	19
Zhang et al.	2024	Cohort (P)	*NS*	≥ 18 years old; sepsis according to SEPSIS-3	<18 years old; pregnancy/lactation; psychiatric disorder; primary brain injury; encephalopathy secondary to organ dysfunction; melanoma, severe burn injury, trauma, neurosurgery; vitreous haemorrhage, eye surgery; lumbar spine disease; history of chronic alcohol/drug abuse; no informed consent; palliative care patients*	101	49/51	62
Torbic et al.	2024	Cohort (P)	Med	18–85 years old; admitted to a medical ICU; confirmed delirium (CAM-ICU); arterial/central/ venous/peripheral line prior to enrolment	Psychiatric disorder; dementia; immunocompromised patients; severe liver dysfunction; active alcohol withdrawal/ admission for drug overdose; antipsychotic use; quetiapine or haloperidol use < 3 days from enrolment; ICU length of stay > 7 days at enrolment; hospital length of stay > 14 days at enrolment	23	57/43	100
Shi et al.	2024	Cohort (R)	*NS*	Sepsis according to SEPSIS-3	Age < 65 years; psychiatric disorder; primary brain injury; dementia; malignancy; palliative care patients*; discharged from the ICU < 48 h; no delirium assessment	2327	55/45	55
Qian et al.	2024	Cohort (R)	*NS*	≥ 18 years old; ICU stay > 24 h; information on serum lactate concentration examined 0–24 h and > 24 h after the ICU admission; not diagnosed with coma or delirium within 24-h stay	Traumatic brain injury; dementia; psychosis; dyslexia or intellectual disability; nervous system diseases; alcohol/drug abuse	7812	57/43	56
Dragoescu et al.	2024	Cohort (P)	*NS*	≥ 18 years old; sepsis diagnosed by the NEWS and/or SOFA score; urinary tract condition or a recent endoscopic or percutaneous urological procedure	< 18 years old; pregnancy; dementia or history of psychiatric disorder; immunocompromised patients; advanced cancer/palliative care patients*; alcohol/drug abuse; antipsychotic use; inability to communicate adequately	76	68/32	49
Brummel et al.	2024	Cohort (P)	Mix	≥ 18 years old; treated for respiratory failure and/or shock (cardiogenic/septic); admitted to a medical or surgical ICU	Acute organ dysfunction for > 72 h; risk of severe preexisting cognitive deficits (e.g. owing to neurodegenerative disease, recent cardiac surgery (within previous 3 months) or suspected anoxic brain injury); palliative care patients*; substantial recent critical illness; blind, deaf or unable to communicate adequately in English; conditions impeding long-term follow-up (active substance abuse, prisoner, homelessness, psychotic disorder or residence > 200 miles from enrolling centre); less than one blood sample; no informed consent	991	61/39	70
Schreiber et al.	2023	Cohort (P)	Med	≥ 18 years old; admitted to a medical ICU; German speaking; written informed consent	Dementia; intellectual disability; hospital length of stay > 7 days prior to ICU admission; no informed consent	343	65/35	35
Plaschke et al.	2023	Cohort (P)	Sur	≥ 18 years old; sepsis according to SEPSIS-3 informed consent	Dementia; stroke; Parkinson’s disease; palliative care patients*	22	73/27	50
Khan et al.	2023 (A)	Cohort (P)	Med	≥ 18 years old; ICU stay ≥ 24 h; confirmed delirium (RASS + CAM-ICU); English speaking; had blood samples collected at two time points	Pregnancy/lactation; psychiatric disorder or history of severe psychiatric disorder; aphasic stroke or traumatic brain injury; severe cognitive impairment or severe dementia; severe visual or auditory disorders; palliative care patients*; alcohol withdrawal delirium; admitted for suicide attempt; history of allergic reaction or contraindication to haloperidol; QTc > 500; previously enrolled in the PMD or De-PMD trial or another study	178	44/56	100
Khan et al.	2023 (B)	Cohort (R)	Med	Admitted to the ICU; acute respiratory failure due to COVID-19; documented positive PCR-test (nasopharyngeal swab) for SARS-CoV-2	< 18 years old; no delirium or coma assessments available; not discharged at the end of the study period; missing laboratory data on CRP, ferritin and D-dimer	197	56/44	100
Huang et al.	2023	Cohort (R)	Med	Had a documented TyG index; delirium assessment during initial ICU admission	< 65 years old; dementia; ICU stay < 24 h	4649**	54/46	47
Smith et al.	2022	Cohort (P)	Mix	≥ 18 years old; critically ill ICU patients	Stroke or traumatic brain injury; palliative care patients*; delirium at ICU admission; no informed consent	119	66/34	34
Smeele et al.	2022	Cohort (P)	Med	PCR-confirmed, severe COVID-19; minimum 7 days of mechanical ventilation; treatment due to persistent acute respiratory distress syndrome	*NS*	31**	74/26	X^a^
Park et al.	2022	Cohort (R)	Mix	Critically ill ICU patients	< 50 years old; no evaluation data recorded, or patient absent at the scheduled time of evaluation; missing data incl. BUN, Cr, or APACHE II score at ICU admission date	7122	60/40	19
Page et al.	2022	Cohort (P)	Mix	Critically ill patients; need of mechanical ventilation within 72 h of admission control-group: adults; scheduled for major elective non-intracranial, non-cardiac surgery; no post-operative delirium; complete dataset	< 18 years old; pregnant/lactation; history of porphyria; severe liver disease or renal impairment not receiving renal replacement therapy; ALAT > 8 times the upper limit of normal range < 72 h of randomization; CK > 10 times upper limit of normal range < 72 h of randomization; uncomplicated elective surgery; ongoing and sustained treatment with any of the following drugs: itraconazole, ketoconazole, HIV protease inhibitors, nefazodone, cyclosporine, amiodarone, verapamil, diltiazem, gemfibrozil or danazol; known allergy to statin drugs; current or recent treatment (< 2 weeks) with statins; statin required for proven indication; contraindication to enteral drug administration; expected discharge < 48 h of admission; palliative care patients*; known participation in investigational medicinal product trials < 30 days; no informed consent; unable to communicate adequately in English	142	37/42	79
Lei et al.	2022	Cohort (P)	Med	≥ 18 years old; admitted to the ICU	< 18 years old or > 85 years old; pregnancy; traumatic brain injury, intracerebral haemorrhage, cerebral infarction; severe circulatory instability; immunological diseases (not specified); malignant hematologic diseases; palliative care patients*; known allergies; drug abuse; ICU stay < 72 h	52	63/37	46
Seo et al.	2021	Cohort (R)	Mix	Delirium group: *NS* non-delirium group: neither delirious state or continuous deep sedation (RASS < −3)	Delirium group: no evaluation data recorded, or patient absent at the scheduled time of evaluation; continuous deep sedation (RASS < −3); no delirium during ICU stay; no baseline data owing to the occurrence of delirium on the day of the initial admission; missing laboratory data of neutrophils, lymphocytes, WBC or CRP level Non-delirium group: discharged from ICU before the 6th ICU day	2384	61/39	47
Steimer et al.	2021	Cohort (P)	Mix	≥ 18 years old; polytrauma (ISS > 16) ± traumatic brain injury; ICU admission < 24 h after trauma; blood drawn < 24 h after admission; informed consent	Dementia; preexisting renal impairment; traumatic or non-traumatic brain injury < 14 days prior to admission; admission > 24 h after trauma; palliative care patients*	48	88/12	40
Pektezel et al.	2021	Cohort (R)	Mix	> 18 years old; admitted to ICU; followed > 48 h	Steroid treatment prior to ICU admission; No data on cortisol levels < 24 h after ICU admission	125	55/45	30
Li et al.	2021	2 cohorts (R + P)	*NS*	Retrospective study arm: ≥ 65 years old; sepsis; admitted to the ICU Prospective study arm: ≥ 65 years old; sepsis according to SEPSIS-3.0; ICU stay > 24 h	Retrospective study arm: history of cognitive impairment; neutropenia at ICU admission; palliative care patients* Prospective study arm: neutropenia; immunodeficiency (HIV, autoimmune disease at active stage, haematological disease, or malignant tumours receiving chemotherapy or glucocorticoids within the previous 3 months); palliative care patients*; no informed consent	R: 1010 P: 247	R:56/44 P:62/38	R: 29 P: 39
Souza-Dantas et al.	2020	Cohort (P)	Mix	≥ 18 years old; admitted to ICU; mechanical ventilation > 48 h	Previous neurologic disorder; palliative care patients*; ventilated > 24 h prior to ICU admission; ventilated after 48 h of ICU admission; readmission; blind or deaf; unable to communicate adequately in Portuguese	629	60/40	20
Wanderlind et al.	2020	Case–control (R)	Mix	> 18 years old; admitted > 72 h; admitted to ICU	Persistent coma; dementia or admission due to neurologic causes (including delirium)	94	65/35	50
Voils et al.	2020	Case–control (R)	Sur	≥ 18 years old; anticipated ICU stay > 48 h; White racial background	Pregnancy/lactation; Alzheimer’s disease or other primary neurologic condition; hepatic encephalopathy or end-stage liver failure; active malignancy; palliative care patients*; visual or hearing impairment; inability to assess delirium (e.g. RASS < −3 or use of neuromuscular blocking medication)	130	65/35	50
Khan et al.	2020	Cohort (P)	Mix	≥ 18 years old; ICU stay ≥ 24 h; confirmed delirium (RASS + CAM-ICU); English speaking; blood drawn at enrolment	Pregnancy/lactation; psychiatric disorder or history of severe psychiatric disorder; aphasic stroke or traumatic brain injury; severe cognitive impairment or severe dementia; severe visual or auditory disorders; palliative care patients*; alcohol withdrawal delirium; admitted for suicide attempt; history of allergic reaction or contraindication to haloperidol; QTc > 500; previously enrolled in the PMD or De-PMD trial or another study	321	44/56	100
Jiang et al.	2020	Cohort (R)	Mix	≥ 18 years old; admitted to the ICU	Brain injury; history of schizophrenia or Parkinson’s disease; coma or profound dementia causing inability to communicate; ICU stay < 24 h	319	50/50	9
Hayhurst et al.	2020	Cohort (P)	Mix	≥ 18 years old; treated for respiratory failure and/or shock (cardiogenic/septic); admitted to a medical or surgical ICU	Acute organ dysfunction for > 72 h; risk of severe preexisting cognitive deficits (e.g. owing to neurodegenerative disease, recent cardiac surgery (within previous 3 months) or suspected anoxic brain injury); palliative care patients*; deceased before hospital discharge; substantial recent critical illness; blind, deaf or unable to communicate adequately in English; conditions impeding long-term follow-up (active substance abuse, prisoner, homelessness, psychotic disorder or residence > 200 miles from enrolling centre); no informed consent; withdrawal from the study; no available biomarker data	427	28/72	77
Cooper et al.	2020	Cohort (P)	Mixed	Critically ill ICU patients; COVID-19 diagnosis ICU controls: critically ill non-COVID-19 patients; admitted to the ICU with a primary diagnosis of respiratory failure from pneumonia Healthy controls: 40–60 years old; volunteer blood donor; no respiratory or neurologic disease	NS ICU controls: acute CNS pathology healthy controls: NS	46	41/59	33
Ozkul et al.	2019	Case–control (P)	*NS*	Cases: delirium; admitted to the ICU controls: no delirium; admitted to the ICU	Idiopathic delirium; history of psychiatric disorders; acute and chronic CNS pathology; endocrine disorders (e.g. thyrotoxicosis); hematologic and immune-related diseases; intoxication; use of anti-inflammatory medication	103	57/43	51
Erikson et al.	2019	Cohort (P)	Mix	Septic shock according to SEPSIS-2.0; possible to assess delirium using CAM-ICU	Psychiatric disorders; acute CNS pathology; chronic alcoholism or other types of encephalopathy; ophthalmological conditions, e.g. age-related macular degeneration, diabetic retinopathy, cataract, angle-closure glaucoma, or eye injury	22	64/36	45
Ehler et al.	2019	Cohort (P)	*NS*	≥ 18 years old; inclusion < 24 h after onset of severe sepsis or septic shock controls: expected ICU stay > 48 h	Preexisting neuromuscular disease, e.g. neuropathies; dementia or history of CNS pathology (e.g. ischemia or haemorrhage); coagulopathy with active bleeding; preexisting renal replacement therapy; high-dose glucocorticoid treatment; palliative care patients*; no informed consent Controls: sepsis; brain dysfunction	25	44/56	64
Simons et al.	2018	Cohort (P)	Mix	≥ 18 years old; expected ICU stay ≥ 24 h; predicted risk of delirium in the ICU > 40% using ‘PRE-DELIRIC’ model; ICU stay ≥ 6 days	Palliative care patients*; inability to assess delirium; remained comatose during the first 7 ICU days; developed delirium after 6 days of ICU admission	50	72/28	70
Zhu et al.	2017	Cohort (P)	*NS*	Women; transferred to the ICU > 24 h postpartum; ICU stay > 7 days controls: 20–50 years old; healthy women; scheduled for healthy examination	Neurological disorder; dementia; history of delirium or depression; immunocompromised patients; chronic inflammatory disease; malignancy; severe visual or auditory disorders; unable to communicate adequately in Chinese	824	0/100	10
Li et al.	2017	Cohort (P)	Mix	≥ 18 years old; admitted to the ICU	Pregnancy; psychiatric disorder; severe dementia; acute CNS pathology; habitual benzodiazepine use; coma or intoxication at ICU admission; delirium at ICU admission (CAM-ICU); ICU admission post cardiopulmonary resuscitation; expected ICU stay < 48 h; severe hearing disability or inability to understand Chinese; leptin measurements unavailable or follow-up information missing; no informed consent	336	71/29	30
Hemauer et al.	2017	Cohort (P)	Mix	≥ 18 years old; treated for respiratory failure and/or shock (cardiogenic/septic); admitted to a medical or surgical ICU	Acute organ dysfunction for > 72 h; risk of severe preexisting cognitive deficits (e.g. owing to neurodegenerative disease, recent cardiac surgery (within previous 3 months) or suspected anoxic brain injury); substantial recent critical illness; palliative care patients*; blind, deaf or unable to communicate adequately in English; conditions impeding long-term follow-up (active substance abuse, prisoner, homelessness, psychotic disorder or residence > 200 miles from enrolling centre); no informed consent	821	51/49	74
Nguyen et al.	2016	Cohort (P)	Mix	Severe sepsis or septic shock according to SEPSIS-2.0; admitted to ICU	< 18 years old; pregnancy; severe psychiatric disorder; dementia; acute CNS pathology; disabling neuromuscular disorder; Parkinson disease; post-cardiorespiratory arrest; severe hypothyroidism; liver cirrhosis; chronic kidney haemodialysis; advanced cancer; alcohol withdrawal; medical treatment interfering with prolactin release; death < 24 h after onset of sepsis	101**	62/38	78
Hughes et al.	2016	Cohort (P)	Mix	≥ 18 years old; treated for respiratory failure and/or shock (cardiogenic/septic); admitted to a medical or surgical ICU	Acute organ dysfunction for > 72 h; risk of severe preexisting cognitive deficits (e.g. owing to neurodegenerative disease, recent cardiac surgery (within previous 3 months) or suspected anoxic brain injury); substantial recent critical illness; palliative care patients*; blind, deaf or unable to communicate adequately in English; conditions impeding long-term follow-up (active substance abuse, prisoner, homelessness, psychotic disorder or residence >200 miles from enrolling centre; no informed consent; lack of reliable endothelial vascular reactivity measurements	134	43/57	70
Anderson et al.	2016	Cohort (P)	Med	Severe sepsis according to SEPSIS-2.0; admitted to medical ICU	Neuro-endocrine cancer; palliative care patients*; transfers from non-participating centres; previous enrolment; primary reason for admission unrelated to sepsis (e.g. cardiac arrest, head injury); Plasma not available; haemolyzed plasma	124^b^	60/40	28
Tomasi et al.	2015	Cohort (P)	Mix	> 18 years; admitted > 24 h	Psychiatric disorder; dementia; CNS disorder; admission due to brain trauma, delirium or other severe neurological condition (e.g. stroke and subarachnoid haemorrhage); inability to assess delirium during ICU stay	77	69/31	49
Zhang et al.	2014	Cohort (P)	Mix	≥ 18 years old; GCS > 10 or RASS ≥ −3; expected ICU stay > 48 h	Acute structural brain damage (e.g. trauma, intracranial haemorrhage, stroke, subdural hematoma); palliative care patients*; delirium at the time of ICU admission (CAM-ICU)	223	63/37	24
Ritter et al.	2014	Cohort (P)	Mix	≥ 18 years old; admitted > 24 h	Admission due to brain trauma, delirium or other severe neurological condition (e.g. stroke and subarachnoid haemorrhage); inability to assess delirium during study period (RASS < −3)	78	69/31	40
Nguyen et al.	2014	Cohort (P)	*NS*	Severe sepsis or septic shock according to SEPSIS-2.0; admitted to ICU	< 18 years old; pregnancy; severe psychiatric disorder; acute CNS pathology; dementia w. disabling neuromuscular disorders; Severe chronic liver or renal failure; drug or alcohol withdrawal; treatment with glucocorticoid or etomidate in the previous 24 h; died from sepsis < 24 h	128	65/35	84
Alexander et al.	2014	Cohort (P)	Mix	21–75 years old; requiring mechanical ventilation for 24–96 h; no preexisting cognitive dysfunction	*NS*	77	47/53	45
Khan et al.	2013	Cohort (P)	Mix	≥ 18 years old; admitted to ICU; confirmed delirium (CAM-ICU); English speaking; two blood samples collected 1 week apart	Pregnancy/lactation; Acute CNS pathology; history of severe psychiatric disorder; admitted with alcohol intoxication; unable to communicate adequately in English	63	38/62	*NS*
Skrobik et al.	2013	Cohort (P)	*NS*	≥ 18 years old; admitted to ICU	Cerebral anoxia; CNS lesion that could cause or mimic coma	99	*NS*	65^c^
Sharma et al.	2012	Cohort (P)	Med	≥ 18 years old	Deaf; unable to communicate adequately in Hindi, English or Punjabi; ICU stay < 24 h;	140	51/49	54
Girard et al.	2012	Cohort (P)	Med	Admitted to medical ICU; mechanic ventilation > 12 h	Profound neurological deficits (e.g. due to large stroke or severe dementia); palliative care patients*; admission post cardiac arrest; current episode of mechanical ventilation > 2 weeks; enrolled in another clinical trial	138	50/50	78
Van den Boogaard et al.	2011	Cohort (P)	Mix	> 18 years old; medical or surgical patient; admitted to ICU	History of severe cognitive impairment; admission due to trauma, post cardiac arrest or neurologic reasons; doubt concerning preexisting cognitive function; not possible to CAM-ICU screen during entire ICU stay	100	53/47	50
McGrane et al.	2011	Cohort (P)	Mix	≥ 18 years old; mechanical ventilation > 24 h; admitted to medical or surgical ICU	Pregnancy/lactation; severe dementia; neurological disease (e.g. previous stroke, cerebral palsy); active seizure; active myocardial ischemia; second- or third-degree heart block; liver disease (Child–Pugh B or C); palliative care patients*; alcohol abuse; benzodiazepine dependency; severe visual or auditory disorders or unable to communicate adequately in English	88	50/50	30
Grandi et al.	2011	Case–control (R)	Mix	Admission > 24 h; admitted to ICU	Previous neurologic disease (e.g. stroke or cerebral palsy); active seizure disorder; severe dementia; inability to assess delirium (severe hearing disability or inability to communicate adequately in Portuguese); RASS < −3 for ≥ 3 days	60	45/55	50
Uguz et al.	2010	Cohort (P)	Med	Acute MI; admitted to coronary ICU	Unconscious, in stupor or coma at initial psychiatric evaluation; transfers from another hospital with ≥ 1 days of hospitalization	212	79/21	6
Pandharipande et al.	2009	Cohort (P)	Mix	≥ 18 years old; mechanical ventilation > 24 h; admitted to medical or surgical ICU	Pregnancy/lactation; severe dementia; neurological disease (e.g. previous stroke, cerebral palsy); active seizures; active myocardial ischemia; second- or third-degree heart block; liver disease (Child–Pugh B or C); palliative care patients*; alcohol abuse; benzodiazepine dependency; severe visual or auditory disorders or unable to communicate adequately in English	97	53/47	*NS*
Pfister et al.	2008	Cohort (P)	*NS*	≥ 18 years old; sepsis, severe sepsis, or septic shock according to SEPSIS-2.0	Intracranial focus of infection; preexisting CNS pathology; delirium attributable to another cause than sepsis; acute or chronic liver failure; uncorrected metabolic derangements	16	63/37	75
Tsuruta et al.	2008	Cohort (P)	*NS*	> 18 years old; admitted to ICU; expected ICU stay > 72 h	Dementia; psychosis; intellectual disability; neuromuscular disease; use of antipsychotics or morphine at enrolment	32	87/13	41
Plaschke et al.	2007	Cohort (P)	*NS*	> 18 years; admitted to the ICU following elective surgery, for emergency reasons, or following trauma	Alzheimer’s disease; depression, schizophrenia; Parkinson disease; history of alcohol or drug abuse; hearing and ⁄ or visual impairment; unable to communicate adequately in German; RASS < −3; sedated to tolerate mechanical ventilation during the 1st and 2nd day following ICU admission	37	73/27	46
Watts et al.	2007	Cohort (R)	*NS*	> 18 years old; no neurological abnormality present on admission; ICU admission < 96 h; informed consent	*NS*	44	59/41	48
Seaman et al.	2006	Cohort (R)	*NS*	Medical patient; admitted to ICU	< 18 to > 85 years old; traumatic brain injury; Stroke; severe circulatory instability; immunological disease; malignant hematologic disease; hepatic encephalopathy; drug or alcohol intoxication or withdrawal; intubated at admission; ICU stay < 72 h	101	*NS*	30

Schematic presentation summarizing the characteristics of the included studies. The study design is categorized as cohort or case control, and as prospective (P) or retrospective (R). The ICU setting is categorized as mixed (mix), medical (med), surgical (sur) or not specified (NS)

*NS* not specified, *R* retrospective, *P* prospective, *‘mix’* mixed ICU, *‘med’* medical ICU, *‘sur’* surgical ICU, *NEWS* National Early Warning Score, *SOFA* Sequential Organ Failure Assessment, *APACHE* Acute Physiology and Chronic Health Evaluation, *CAM-ICU* Confusion Assessment Method for the Intensive Care Unit, *RASS* Richmond Agitation Sedation Scale, *QTc* corrected QT-interval, *PCR* polymerase chain reaction, *ALAT* alanine aminotransferase, *CK* creatine kinase

^*^Most studies used varying terminology to describe exclusion of patients in moribund or end-of-life conditions. For the purpose of this review, the term ‘palliative care patients’ was applied to encompass the following classifications: death within 24 or 48 h, withdrawal of life-sustaining treatment, do-not-resuscitate (DNR) or do-not-intubate (DNI) orders, terminal illness or terminal state, explicit reference to palliative care or comfort measures only, patients described as moribund, and those with anticipated treatment withdrawal or a life expectancy of less than 48, 24, or 12 h

^**^’Number of patients’ refers only to those included in the ‘study cohort’ with data on delirium. Number of patients from age-matched control groups or included cohorts not analysed in relation to delirium, as well as those from external validation cohorts, are not included

^a^Reported as ‘delirium 2 days after extubation: 18 Y/4 N (Yes/No)’ and ‘delirium 5 days after extubation 14 Y/7 N (Yes/No)’

^b^Delirium was only assessed in 108 of these 124 patients

^c^Reported as the total number of ‘patients w. delirium and coma’ and ‘patients w. delirium’. Of these 64 patients only 36 had blood samples drawn for analyses and only 7 of those patients had ‘only delirium’

### Delirium outcomes

Table 2 presents details on delirium outcomes, assessment methods, screening frequencies, delirium definitions, and reported outcome types across studies.

**Table 2:** Delirium assessment and outcomes

First author (year)	Assessment tool			Daily assessments	Definition of delirium*	Reported delirium outcomes						
	Screening	Severity	Subtype			Occurrence**	Duration	Time to onset	Severity	‘Delirium-free’ or ‘delirium-/coma-free’ days	Composite outcome: delirium + coma	Subtype
Pham (2025)	CHART-DEL-ICU	–	–	–	Delirium diagnosis documented in medical record	X	–	X	–	–	–	–
Fayssoil (2025)	CAM	–	–	1 + upon fluctuations in mental status	1 pos. CAM-ICU	X	–	–	–	–	–	–
Zhang (2025)	CAM-ICU	–	–	–	1 pos. CAM-ICU	X	–	–	–	–	–	–
Wang (2025)	CAM-ICU	–	–	–	1 pos. CAM-ICU	X	–	–	–	–	–	–
Viegas (2024)	CAM-ICU	–	–	–	1 pos. CAM-ICU or comprehensive review of clinical notes	X	–	–	–	–	–	–
Zhang (2024)	CAM-ICU	–	–	2	1 pos. CAM-ICU	X	–	–	–	–	–	–
Torbic (2024)	CAM-ICU	–	–	3	1 pos. CAM-ICU	N/A^a^	–	–	–	N/A^a^	–	–
Shi (2024)	CAM-ICU	–	–	–	1 pos. CAM-ICU	X	–	–	–	–	–	–
Qian (2024)	CAM-ICU	–	–	–	1 pos. CAM-ICU	X	–	–	–	–	–	–
Dragoescu (2024)	CAM-ICU	–	–	1	1 pos. CAM-ICU	X	–	–	–	–	–	–
Brummel (2024)	CAM-ICU	–	–	2	1 pos. CAM-ICU	X	X	–	–	X	–	–
Schreiber (2023)	CAM-ICU	–	–	2	1 pos. CAM-ICU	X	X	–	–	–	–	–
Plaschke (2023)	ICDSC + CAM-ICU	–	–	3	1 pos. CAM-ICU	X	–	–	–	–	–	–
Khan (2023 A)	CAM-ICU	CAM-ICU-7	–	2	1 pos. CAM-ICU	N/A^a^	–	–	X	X	–	–
Khan (2023 B)	CAM-ICU	–	–	2	1 pos. CAM-ICU	–	–	–	–	–	–	–
Huang (2023)	CAM-ICU + ICDSC	–	–	–	*NS*	X	–	–	–	–	–	–
Smith (2022)	CAM-ICU	–	–	3 + upon fluctuations in mental status	*NS*	X	–	–	–	–	–	–
Smeele (2022)	CAM-ICU + CHART-DEL	DOS	–	–	*NS*	X	X	–	X	–	–	–
Park (2022)	CAM-ICU	K-DRS-98	DMSS	1	1 pos. CAM-ICU + DSM-5 criteria	X	–	X	–	–	–	X
Page (2022)	CAM-ICU	–	–	–	1 pos. CAM-ICU	X	X	–	–	–	–	–
Lei (2022)	CAM-ICU	–	–	–	*NS*	X	–	–	–	–	–	–
Seo (2021)	CAM-ICU	K-DRS-98	DMSS	1	1 pos. CAM-ICU + DSM-5 criteria	X	–	X	X	–	–	X
Steimer (2021)	nuDesc	–	–	3	*NS*	X	–	–	–	–	–	–
Pektezel (2021)	CAM-ICU (Turkish version)	–	–	3	*NS*	X	–	–	–	–	–	–
Li (2021)	CAM-ICU (simplified Chinese version)	–	–	2 + upon fluctuations in mental status	1 pos. CAM-ICU	X	–	–	–	–	–	–
Souza-Dantas (2020)	CAM-ICU (Brazilian-Portuguese version)	–	–	1	*NS*	X	X	–	–	–	X	–
Wanderlind (2020)	CAM-ICU	–	–	2	*NS*	X	X	–	–	–	–	–
Voils (2020)	CAM-ICU	–	RASS	2	1 pos. CAM-ICU	X	–	X	–	–	–	X
Khan (2020)	CAM-ICU	CAM-ICU-7	–	2	*NS*	N/A^a^	X	–	X	X	–	–
Jiang (2020)	CAM-ICU	–	–	2	*NS*	X	–	–	–	–	–	–
Hayhurst (2020)	CAM-ICU	–	–	2	1 pos. CAM-ICU	X	X	–	–	–	–	–
Cooper (2020)	ICDSC	–	–	*NS*	≥ 4 points on ICDSC	X	–	–	–	–	–	–
Ozkul (2019)	CAM-ICU	–	–	–	DSM-4 criteria	X	–	–	–	–	–	X
Erikson (2019)	CAM-ICU (Finnish version)	–	–	–	*NS*	X	–	–	–	–	–	–
Ehler (2019)	CAM-ICU + ICDSC	–	–	*NS*	1 pos. CAM-ICU	X	–	–	–	–	–	–
Simons (2018)	CAM-ICU	–	RASS	3	1 pos. CAM-ICU	X	–	X	–	–	–	X
Zhu (2017)	CAM-ICU	–	–	1	–	X	–	–	–	–	–	–
Li (2017)	CAM-ICU	–	–	2	1 pos. CAM-ICU	X	–	–	–	–	–	–
Hemauer (2017)	CAM-ICU	–	–	1	1 pos. CAM-ICU	X	X	–	–	–	–	–
Nguyen (2016)	CAM-ICU	–	RASS	2	Pos. CAM-ICU for 2 consecutive days	X	X	–	–	–	–	X
Hughes (2016)	CAM-ICU	–	–	2	1 pos. CAM-ICU	X	X	–	–	X	–	–
Anderson (2016)	CAM-ICU	–	–	*NS*	1 pos. CAM-ICU or 1 progress note w. documented delirium diagnosis	X	–	–	–	X	–	–
Tomasi (2015)	CAM-ICU	–	–	2	≥ 1 pos. CAM-ICU screening criterion	X	X	–	–	X	–	–
Zhang (2014)	CAM-ICU	–	RASS	3	*NS*	X	–	–	–	–	–	X
Ritter (2014)	CAM-ICU	–	–	2	≥ 1 pos. CAM-ICU screening criterion	X	–	–	–	–	–	X
Nguyen (2014)	CAM-ICU	–	RASS	2	Pos. CAM-ICU for 2 consecutive days	X	–	–	–	–	X	X
Alexander (2014)	CAM-ICU	–	–	1	1 pos. CAM-ICU	X	X	–	–	X	–	–
Khan (2013)	CAM-ICU	–	–	2	1 pos. CAM-ICU	N/A^a^	X	–	–	–	–	–
Skrobik (2013)	ICDSC	–	–	3	≥ 4 points on ICDSC	X	–	X	–	–	–	–
Sharma (2012)	DSM-4 criteria	DRS-R-98	RASS	1	*NS*	X	–	–	X	–	–	X
Girard (2012)	CAM-ICU	–	–	1	*NS*	X	X	–	–	–	–	–
Van den Boogaard (2011)	CAM-ICU	–	–	3	1 pos. CAM-ICU	X	–	–	–	–	–	–
McGrane (2011)	CAM-ICU	–	–	1	1 pos. CAM-ICU	X	–	–	–	X	–	–
Grandi (2011)	CAM-ICU	–	–	2	1 pos. CAM-ICU	N/A^a^	X	X	–	–	–	–
Uguz (2010)	DSM-4 criteria	DRS	*NS*	1	DSM-4 criteria	X	X	X	X	–	–	X
Pandharipande (2009)	CAM-ICU	–	–	–	1 pos. CAM-ICU	–	–	–	–	–	–	–
Pfister (2008)	CAM-ICU	–	–	1	*NS*	X	–	–	–	–	–	–
Tsuruta (2008)	CAM-ICU (Japanese version)	–	–	1	1 pos. CAM-ICU	X	–	–	–	–	–	–
Plaschke (2007)	CAM-ICU (German version)	–	–	1	1 pos. CAM-ICU	X	–	–	–	–	–	–
Watts (2007)	ICDSC	–	–	2	*NS*	X	–	–	–	–	–	–
Seaman (2006)	CAM	–	–	–	1 pos. CAM	X	–	–	–	–	–	–

An overview of included studies summarizing the delirium assessment methods, frequency of assessments, definition of delirium and reported delirium-related outcomes. An empty field represents that the given method, frequency, definition or outcome was not reported

*NS* not specified, *N/A* not applicable, *pos* positive, *CAM-ICU* Confusion Assessment Method for the Intensive Care Unit, *ICDSC* Intensive Care Delirium Screening Checklist, *CAM-ICU-7* Confusion Assessment Method for the Intensive Care Unit-7, *DOS* Delirium Observation Screening Scale, *DSM-5* Diagnostic and Statistical Manual of Mental Disorders, Fifth Edition, *(K-)DRS-98* (Korean version of) Delirium Rating Scale Revised-98, *NuDesc* nursing delirium screening scale, *CHART-DEL* Chart-Based Delirium Identification instrument, *DMSS* Delirium Motor Subtype Scale, *RASS* Richmond Agitation Sedation Scale

^*^Studies were categorized as ‘not specified’ if no explicit definition of delirium was provided, e.g. if the definition was reported as ‘based on the CAM-ICU’ but no description of a specific number of screenings required to establish the diagnosis was given

^**^Represents either incidence or prevalence, as reported by the original study authors

^a^Not applicable because of study design, e.g. case–control study or a cohort exclusively including delirious patients

The most frequently reported outcome was delirium prevalence (*n* = 54), followed by duration (*n* = 17), subtype classification (*n* = 11), delirium/coma-free days (*n* = 8), time to onset (*n* = 8), and delirium severity (*n* = 6). Delirium definition varied markedly across studies with more than six distinct definitions applied. The majority defined delirium as at least one positive CAM-ICU screening during ICU stay, but 17 studies provided no explicit definition (see Table 2 for details and references). Figure 2 illustrates the distribution of outcomes and definitions.

On average, delirium was assessed twice daily, although frequency varied: 44 studies conducted daily evaluations and 17 provided no explicit statement. Among studies reporting daily assessments, six strategies were identified: once (*n* = 14), twice (*n* = 19) or three times daily (*n* = 8), with three additionally applying event-triggered reassessment (see Table 2 for references). Outcome ascertainment relied on sixdifferent validated clinical assessment scales [13, 14, 98–101], predominantly ‘Confusion Assessment Method for the Intensive Care Unit’ (CAM-ICU) [13], with a few articles combining the scales (see Table 2 for references). Additional scales were applied to assess delirium severity and motor subtype [102–108] (see Table 2).

Follow-up periods for delirium assessment varied widely. Nineteen studies did not clearly specify follow-up [37–39, 43, 44, 46, 47, 56, 58, 62–64, 66, 79, 87, 89–91, 96]. Thirteen assessed delirium until ICU discharge [41, 50, 53, 55, 57, 60, 70, 76, 81, 83, 88, 94, 97], five until hospital discharge [48, 49, 54, 78, 92], and seven used predefined maximum durations ending at ICU/hospital discharge, death, or fixed limits (14–30 days) [40, 42, 45, 71, 73, 74, 80]. Fifteen applied fixed periods (48 h [85, 95], 72 h [75], 5 d [77, 86], 7 or 8 d [67, 93], 12 d [82, 84], 15 d [72], 28 d [51, 68] or 30 d [59, 61, 69]), one used four predefined time points (day 1, 3, 7, and 28) [65], and one continued follow-up after ICU discharge (28 d) [52]. Overall, 21 distinct follow-up strategies were identified, underscoring pronounced methodological heterogeneity.

Additional patient characteristics and outcomes are provided in Supplementary Table 1 (in Supplementary Materials 1).

### Biomarkers

A total of 153 serum biomarkers were examined, of which 101 (66%) demonstrated statistical significance. Fifty-eight (38%) biomarkers were significantly associated with delirium in unadjusted analyses, and 43 (28%) in adjusted analyses. Furthermore, 40 (26%) biomarkers were statistically associated with delirium in at least two studies. Figure 2 illustrates the distribution of statistical analysis approaches and biomarker categories assessed.

Biomarker sampling strategies varied markedly across studies. Sampling most commonly occurred at ICU admission, study enrolment, or within 24 h (*n* = 21) [39, 41, 43, 44, 47, 48, 55, 56, 58–60, 63, 67, 71–73, 82, 86, 91, 92, 96], while others employed broader early sampling windows (< 48 or < 72 h; *n* = 5) [37, 64, 68, 85, 95]. Fourteen studies combined sampling at admission with predefined subsequent fixed time points [40, 42, 45, 51, 52, 54, 61, 62, 65, 66, 74, 75, 78, 84], six applied repeated daily sampling over a fixed period [46, 70, 76, 80, 87, 93], and six used event- or disease-dependent strategies [38, 53, 57, 79, 81, 83]. Two studies relied on routine clinical practice [49, 88], and seven did not specify timing of the sampling [50, 69, 77, 89, 90, 94, 97]. Overall, 27 studies (44%) used a single sampling time point, 12 (20%) sampled twice, 13 (21%) sampled three or more times, and nine (15%) did not report sampling frequency (see Table 3 for references).

**Table 3:** Biomarkers

Author (year)	Investigated biomarkers	Timing of the sampling	Sampling site	Significant association		Main study conclusions
				Unadjusted	Adjusted	
Pham (2025)	BDNF, NfL, Ng, S100β, CHI3L1, TREM2, GFAP, creatinine	< 24 h after ICU admission	*NS*	BDNF, NfL	BDNF, NfL	Increased NfL levels were associated with delirium occurrence Decreased BDNF levels were associated with delirium occurrence
Fayssoil (2025)	CRP, NT-proBNP, troponin, creatinine, glucose, sodium	*NS*	*NS*	CRP, NT-proBNP, troponin	CRP	Increased CRP levels were associated with delirium occurrence
Zhang (2025)	TyG-index, TyG-AVG	*NS*	*NS*	TyG index, TyG-AVG	TyG index, TyG-AVG	Elevated TyG index and TyG-AVG were associated with delirium occurrence
Wang (2025)	WBC, platelets, lymphocytes, monocytes, neutrophils, NLR, LMR, PLR, Hb, albumin, creatinine, glucose	*NS*	*NS*	WBC, platelets, lymphocytes, monocytes, neutrophils, NLR, LMR, Hb, albumin, creatinine, glucose	PLR, NLR, LMR	Increased neutrophil-to-lymphocyte ratio and platelet-to-lymphocyte ratio were associated with delirium occurrence Decreased lymphocyte-to-monocyte ratio were associated with delirium occurrence
Viegas (2024)	Serum spectral bands	Morning after ICU admission	Peripheral blood	Bands at 2853, 2926, 2961, 1470 and 1658 cm^−1^	Bands at 2912, 1149, 942, 1546, 1268, 987 and 1041 cm^−1^	Specific serum spectral bands were associated with delirium occurrence
Zhang (2024)	S100β, NSE and BNIP3L WBC, neutrophils, lactates, PCT, ALAT, ASAT, albumin, creatinine, BUN, CRP	< 72 h of enrolment	*NS*	WBC, neutrophils, lactate, PCT, NSE, S100β	Neutrophils, S100β	Increased neutrophils and plasma S100β levels were associated with delirium occurrence
Torbic (2024)	IL-6, IL-8	Prior to 1st dose of antipsychotic medication (AP) + 4–8 h after enrolment or 1st AP dose + 22–28 h after enrolment or AP administration	*NS*	None		No significant finding
Shi (2024)	LMR, WBC, monocytes, neutrophils, platelets, PLR, lymphocytes, NLR, BUN, creatinine, ALAT, ASAT, bilirubin, albumin, INR, PT, PTT, Hb, glucose, anion gap, HCO_3_, Na, Ca, K, and lactate	1st day of ICU admission	*NS*	WBC, monocytes, neutrophils, platelets, lymphocytes, LMR, creatinine, BUN, ALAT, ASAT, bilirubin, anion gap, Na, HCO_3_,	LMR, bilirubin, anion gap, Na	Increased anion gap and sodium levels were associated with delirium occurrence Increased lymphocyte-to-monocyte ratio (LMR) and bilirubin levels were associated with decreased delirium occurrence
Qian (2024)	S-lactate, lactate clearance rate, WBC, RDW, creatinine, BUN, INR, PT, platelets, Na, K, Cl, HCO_3_, pH, glucose	< 24 h after ICU admission (T0 lactate) + > 24 h after admission (T1 lactate)	Arterial blood gas, site unknown	pH, WBC, RDW, platelets, creatinine, INR, BUN, HCO_3_, Na	s-lactate, lactate clearance rate	Increased s-lactate levels were associated with delirium occurrence Increased lactate clearance rate was associated with decreased delirium occurrence
Dragoescu (2024)	NLR, WBC, neutrophils, lymphocytes, platelets, lactate, creatinine, bilirubin, ESR, CRP, PCT	At admission	Peripheral venous blood	NLR	Not reported	Increased neutrophil-to-lymphocyte ratio was associated with delirium occurrence
Brummel (2024)	CRP, IFN-γ, IL-1β, IL-6, IL-8, IL-10, IL-12, MMP-9, TNF-α, TNFR1, protein C	At study enrolment (“day 1”), day 3 and 5	*NS*	Not reported	IL-6, IL-8, IL-10, TNF-α, TNFR1, protein C	Increased IL-6, IL-8, IL-10, TNF-α and TNFR1 levels were associated with delirium occurrence Decreased protein C levels were associated with delirium occurrence Increased IL-6, IL-8 and IL-10 levels were associated with greater delirium duration Decreased protein C levels were associated with greater delirium duration
Schreiber (2023)	CDT, Anttila index Leukocytes, Hb, platelets, CRP, PCT, Na, K, Ca, phosphate, creatinine, urea, bilirubin, γGT, ASAT, ALAT, amylase, creatine kinase, LDH, INR, protein, albumin, lactate	At ICU admission	*NS*	Platelets, CRP, PCT, creatinine, urea, γ-GT, ASAT, INR, protein, albumin, CDT, Anttila index	CDT, Anttila index	Increased CDT levels and Anttila index were associated with delirium occurrence Increased CDT levels and Anttila index were associated with greater delirium duration
Plaschke (2023)	Specified protein expression, pH, glucose, CRP, HCO_3_, platelets, creatinine, bilirubin, lactate, leucocytes	At study enrolment	*NS*	PON1, THBS1, FGG, IgHV3, C1QC	Not reported	Increased expression of PON1, THBS1 and FGG were associated with delirium occurrence Lower expression of IgHV3 and C1QC were associated with delirium occurrence NB: after FDR correction for multiple testing no significant changes were obtained
Khan (2023) (A)	IL-1, IL-6, IL-8, IL-10, TNF-α, CRP, IGF-1, S-100β	< 24 h of study enrolment (“day 1”) + on day 8	*NS*	IL-6, IL-10, CRP, TNF-α, IGF	Not reported	Increased IL-6, CRP and IGF levels weakly correlated with delirium severity Increased IL-6, IL-10, TNF-α, IGF and CRP levels weakly correlated with greater delirium duration
Khan (2023) (B)	CRP, D-dimer, ferritin	Once daily, up to 14 days	*NS*	Not reported	D-dimer, ferritin	Increased D-dimer and ferritin were associated with delirium occurrence Increased CRP was associated with increased delirium severity (survivors only)
Huang (2023)	TyG index, WBC, RBC, BUN, creatinine, glucose, triglyceride levels, albumin, platelets, Na, K, INR	< 24 h after ICU admission	*NS*	WBC, RBC, BUN, creatinine, glucose, triglyceride levels	TyG index	Increased TyG index were associated with delirium occurrence
Smith (2022)	CCL2, CCL3, CXCL1, CXCL10, G-CSF, GM-CSF, IL-1α, IL-1β, IL-1RA, IL-2, IL-4, IL-5, IL-6, IL-8, IL-10, IL-12, IL-17A, IL-18, and TNF-α pH, albumin, BUN, glucose, WBC, lactate, ionized Ca, Ca, Na, creatinine, bilirubin, haematocrit	< 12 h after ICU admission	*NS*	WBC, BUN, glucose, TNF-α, IL-1RA, IL-6, IL-8, IL-10, IL-18, CCL2, CCL3, CXCL1, CXCL10	Not reported	Increased glucose, WBC, BUN, TNF-α, IL-1RA, IL-6, IL-8, IL-10, IL-18, CCL2, CCL3, CXCL1 and CXCL10 levels were associated with delirium occurrence Decreased albumin and pH levels were associated with delirium occurrence
Smeele (2022)	NfL	“Routine clinical practice”	*NS*	NfL	None	Increased NfL levels correlated with delirium duration
Park (2022)	BCR, BUN, creatinine	*NS*	*NS*	BCR, BUN, creatinine	BCR	Increased blood urea creatinine ratio was associated with hypoactive delirium NB: results did not apply to hyperactive or mixed-type delirium
Page (2022)	NfL	Prior to statin administration (“day 1”) + on day 3, 7, 14 and 28	*NS*	NfL	NfL	Increased NfL levels were associated with delirium duration
Lei (2022)	T lymphocytes *(CD3*^+^*CD19*^*−*^*)*, B lymphocytes *(CD3*^*−*^*CD19*^*−*^*)*, classical monocyte cell *(CD3*^*−*^*CD19*^*−*^*CD14*^*hi*^*CD16*^*−*^*)*, intermediate monocytes *(CD3*^*−*^*CD19*^*−*^*CD14*^*hi*^*CD16*^+^*)*, nonclassical monocytes *(CD3*^*−*^*CD19*^*−*^*CD14*^*lo*^*CD16*^+^*)*, natural killer cell *(CD3*^*−*^*CD19*^*−*^*CD14*^*−*^*CD16*^+^*CD56*^+^*)*	Day 1, 3 and 5 after ICU admission	Peripheral blood	CD14^hi^CD16^−^ classical monocyte	Not reported	Decreased CD14^hi^CD16^−^ classical monocyte counts were associated with delirium occurrence
Seo (2021)	NLR, CRP, WBC, neutrophils, lymphocytes	Day of ICU admission + on the day of initial delirium onset	*NS*	NLR, CRP, lymphocyte counts	CRP	Increased neutrophil-to-lymphocyte ratio and CRP levels from baseline to delirium onset were associated with delirium occurrence Decreased lymphocyte counts from baseline to delirium onset were associated with delirium occurrence Increased CRP levels were associated with delirium occurrence (non-hypoactive vs. hypoactive delirium)
Steimer (2021)	HO1 and PER2 mRNA expression, bilirubin	< 24 h of admission (morning hours) + 1 week after trauma	Arterial or venous catheters, site unknown	HO1 and PER2 mRNA expression	Not reported	Increased HO1 and PER2 mRNA expression levels were associated with delirium occurrence
Pektezel (2021)	Plasma cortisol, Hb, leukocytes, creatine, BUN, albumin, Na	At admission	*NS*	None		No significant finding
Li (2021)	Retrospective study arm: NLR, PCT, CRP, BDG, neutrophils, lymphocytes, lactate Prospective study arm: NLR, PCT, CRP, BDG, neutrophils, lymphocytes, B-lymphocytes, T-lymphocytes, CD4^+^ T, CD8^+^ T, NK cells, C3, C4, IgA, IgG, IgM	Retrospective study arm: at admission, 24 h and 48 h after admission Prospective study arm: at ICU admission + at routine examination	Peripheral blood	Retrospective study arm: PCT, lymphocytes, NLR Prospective study arm: PCT, lymphocytes, NLR, NK cells	Retrospective study arm: NLR, lymphocytes Prospective study arm: *not reported*	Retrospective study arm: increased neutrophil-to-lymphocyte ratio and lymphocyte counts were associated with delirium occurrence Prospective study arm: increased PCT levels, lymphocyte counts and NK cell counts were associated with delirium occurrence Decreased neutrophil-to-lymphocyte ratio was associated with delirium occurrence
Souza-Dantas (2020)	CRP-ratio, baseline CRP	At ICU admission + once daily for 7 consecutive days	*NS*	Baseline CRP	CRP-ratio	Increased CRP-ratio were associated with delirium duration
Wanderlind (2020)	IL-1β, Ng	< 24 h after ICU admission (“D1”) + on the day of delirium diagnosis or 48 h after ICU admission (“D2”)	Venous blood, site unknown	IL-1β, Ng	Not reported	Increased IL-1β level at D1 and increased Ng levels at D2 were associated with delirium occurrence
Voils (2020)	Global metabolomics + targeted tryptophan pathway metabolomics analysis	< 24 h after hospital admission	*NS*	Not reported	Global analysis: ascorbic acid Targeted analysis: kynurenic acid, tryptophan, kynurenine-to-tryptophan ratio	Decreased tryptophan levels were associated with delirium occurrence Increased tryptophan metabolites, ascorbic acid level and kynurenine-to-tryptophan ratio were associated with delirium occurrence
Khan (2020)	IL-6, IL-8, IL-10, TNF-α, CRP, S100β, IGF-1	< 24 h after enrolment (between 9 and 11 a.m.)	Venous blood, site unknown	Not reported	IL-6, IL-8, IL-10, TNF-α, CRP, S100β	Increased IL-6, -8, -10, TNF-α, CRP and S100β levels were associated with greater delirium duration Increased IL-6, -8, -10, TNF-α and CRP levels were associated with higher mean delirium severity
Jiang (2020)	PLR, glucose, platelets, albumin, Na, CRP, creatinine, RBC, WBC, NLR, neutrophils, lymphocytes	< 24 h after ICU admission	*NS*	PLR, s-albumin, s-glucose, platelet count	PLR	Increased platelet-to-lymphocyte ratio was associated with delirium occurrence
Hayhurst (2020)	UCHL-1, BDNF	At study enrolment + < 72 h after onset of organ failure and ICU admission	*NS*	Not reported	UCHL-1	Increased UCHL-1 levels were associated with decreased delirium occurrence
Cooper (2020)	GFAP, t-tau, NfL, UCHL-1	As routine care + on day 1–10, day 14 and day 21 after ICU admission	*NS* + arterial blood, site unknown	GFAP, NfL, UCHL-1	Not reported	Increased GFAP, UCHL-1 and NfL levels were associated with delirium occurrence (only in COVID-19 ICU patients)
Ozkul (2019)	IL-6, NSE, S100β	< 12 h of ICU admission	*NS*	IL-6	Not reported	Increased IL-6 levels were associated with delirium occurrence Lower IL-6 levels were associated with delirium occurrence (hypoactive delirium vs. non-hypoactive delirium)
Erikson (2019)	S100β, NSE, HAB42, SUBP, CRP, PCT, IL‐6, IL‐17, TNF‐α, creatinine, lactate, PCT, CRP	15–35 h after ICU admission	*NS*	S100β, IL-6	Not reported	Increased S100β and IL-6 levels associated with delirium occurrence
Ehler (2019)	NfL, NfH	On study days 1, 3 and 7	*NS*	NfL, NfH	Not reported	Greater increase in NfL levels from day 1 to 7 were associated with delirium occurrence Peak NfL levels correlated with delirium occurrence
Simons (2018)	TNF-α, IL-6, IL-1β, IL-10, MCP-1, adiponectin, neopterin, t-tau, Aβ_1–42_, Aβ_1–40_, ratio Aβ_1–40/42_, ratio tau/Aβ_1–42_	Morning after ICU admission (8 am on “day 1”) + on day 2, 4 and 6	Indwelling arterial catheter	t-tau, ratio tau/A_β1–42_, neopterin, IL-10	Adiponectin	No difference in biomarker levels between patients with and without delirium one day prior to or following the onset of delirium Greater adiponectin levels over time were associated with delirium occurrence Increased t-tau levels and ratio tau/Aβ_1–42_ were associated with delirium occurrence (hypoactive vs. no delirium) Increased neopterin and IL-10 levels were associated with delirium occurrence (hypoactive vs. mixed-type delirium)
Zhu (2017)	Galectin-3, S100β, CRP	At ICU admission + at study enrolment	Venous blood, site unknown	Galactin-3, S100β, CRP	Not reported	Increased galactin-3, S100β and CRP levels were associated with delirium occurrence
Li (2017)	Leptin	Day after ICU admission (between 6 and 7 a.m., after overnight fasting)	Venous blood, site unknown	Leptin	Leptin	Decreased plasma leptin levels were associated with delirium occurrence
Hemauer (2017)	Daily lowest haemoglobin (If patients had only one measurement per day, daily lowest hemoglobin represents their single hemoglobin measurement for that day)	*NS*	*NS*	None		No significant finding
Nguyen (2016)	Prolactin, creatinine, CRP	6 and 12 h after ICU admission + then once in the morning for the next 3 days	*NS*	Prolactin, creatinine	Prolactin	Increased prolactin levels in combined effect with age were associated with delirium occurrence Prolactin levels correlated with delirium duration
Hughes (2016)	PAI-1, E-selectin, Ang-2, S100β	At study enrolment	*NS*	Not reported	PAI-1, E-selectin, S100β	Increased PAI-1, E-selectin and S100β levels were associated with greater delirium duration
Anderson (2016)	NSE	At or just prior to ICU admission	*NS*	NSE	NSE	Increased NSE levels were associated with delirium occurrence Increased NSE levels at admission were associated with greater delirium duration
Tomasi (2015)	s-AChE activity, serotonin	< 24 h after ICU admission	*NS*	s-AChE	None	Increased s-AChE levels correlated with greater delirium duration
Zhang (2014)	ΔCRP	At ICU admission + 24 h after ICU admission	*NS*	CRP	ΔCRP	Increase in CRP levels during admission were associated with delirium occurrence Increased CRP levels were associated with delirium occurrence (hypoactive and mixed-type delirium vs. no delirium)
Ritter (2014)	TNF-α, STNFR1, STNFR2, adiponectin, IL-1β, IL-6, IL-10	< 12 h of ICU admission	*NS*	STNFR1, STNFR2, adiponectin, IL-1β	STNFR1, STNFR2, adiponectin, IL-1β	Increased STNFR1, STNFR2, adiponectin and IL-1β levels were associated with delirium occurrence
Nguyen (2014)	s-cortisol, S100β	6–12 h after hemodynamic stabilization in the ICU + in the morning for 4 consecutive days	*NS*	s-cortisol, S100β	s-cortisol	Increased cortisol levels were associated with occurrence of brain dysfunction (delirium and coma)
Alexander (2014)	s-apoE, IL-6, IL-8 and IL-10	*NS*	Arterial or central venous catheters	IL-6	Not reported	Increased IL-6 levels were associated with delirium occurrence
Khan (2013)	S100β	Day 1 and 8 of enrolment (between 9 and 11 a.m.)	*NS*	S100β	None	Increased S100β levels on both day 1 and day 8 were associated with delirium duration*
Skrobik (2013)	TNF-α, IL-17, IL-8, MCP-1, IL-1RA, MIP-1β, and IL-10	< 24 h after “the clinical state of delirium, coma or neither”	*NS*	IL-6	Not reported	Increased IL-6 levels were associated with delirium occurrence
Sharma (2012)	Na, K, pH, uric acid, urea, albumin, creatinine, bilirubin, ASAT, ALAT	*NS*	*NS*	Uric acid, ALAT, pH, albumin	Not reported	Increased uric acid and ALAT were associated with delirium occurrence Decreased pH and albumin were associated with delirium occurrence
Girard (2012)	CRP, MMP-9, MPO, NGAL, STNFR-1, D-dimer, protein C, PAI-1, VWF	Morning after enrolment (or latest < 2 days after enrolment) + on study day 5	Venous blood, site unknown	Not reported	sTNFR1, protein C, MMP-9	Increased sTNFR-1 levels were associated with delirium occurrence Decreased protein C levels were associated with delirium occurrence Increased MMP-9 levels were associated with decreased delirium occurrence
Van den Boogaard (2011)	TNF-α, IL-1b, IL-6, IL-8, IL-17, IL-18, MIF, IL-1RA, IL-10, MCP-1, HNP-1, CRP, PCT, s-cortisol, Aβ_1-42_, Aβ_1-40_, Aβ_N-42_ and Aβ_N-40_, S100β, tau, ratio tau/Aβ_1-42_, ratio Aβ_1-42/40_, ratio Aβ_N-42/40_, ratio Aβ_1-42/N-42_, ratio Aβ_1-40/N-40_	< 24 h after the 1st positive CAM-ICU (between 6 and 10 a.m.)	Indwelling arterial catheter	TNF-α, IL-6, IL-8, IL-18, IL-1Ra, IL-10, MCP-1, MIF PCT, s-cortisol, Aβ_1-40_, Aβ_N-40_, ratio Aβ_1-42/40_, ratio Aβ_N-42/40_	IL-8, IL-10 ratio Aβ_1-42/40_	Increased IL-8 levels were associated with delirium occurrence (inflamed patients only) Increased ratio Aβ_1-42/40_ was associated with decreased delirium occurrence (non-inflamed patients only) Increased IL-10 levels were associated with delirium occurrence (non-inflamed patients only)
McGrane (2011)	PCT, CRP	< 24 h after enrolment	*NS*	Not reported	PCT	Increased baseline PCT levels were associated with greater delirium duration
Grandi (2011)	NSE, S100β, s-BDNF	Cases: at ICU admission + on the day before delirium Controls: at ICU admission + at a subsequent time point corresponding to a measurement from a matched delirious patient	Venous blood, site unknown	NSE, s-BDNF	Not reported	Increased serum NSE and BDNF levels were associated with delirium occurrence Increased serum NSE and BDNF levels were also associated with earlier delirium occurrence (< 3 days vs. > 4 days after ICU admission)
Uguz (2010)	Total cholesterol, LDL, HDL, triglycerides, MB-CK, troponin-I, ASAT, ALAT, Na, K, Ca, glucose	At ICU admission	*NS*	K, glucose	K	Increased potassium levels were associated with delirium occurrence
Pandharipande (2009)	Trp/LNAA ratio, Phe/LNAA ratio, Tyr/LNAA ratio	On study days 1 and 3	*NS*	Not reported	Trp/LNAA ratio, Tyr/LNAA ratio	High and extremely low plasma tryptophan/LNAA and tyrosine/LNAA ratios were associated with transitioning to delirium
Pfister (2008)	CRP, IL-6, S100β, cortisol	After stabilization, but < 48 h after admission	*NS*	CRP, S100β, cortisol	Not reported	Increased CRP, S100β and cortisol levels were associated with delirium occurrence
Tsuruta (2008)	CRP, PAA	< 24 h after admission (non-intubated patients and < 24 h after extubation for intubated patients)	*NS*	CRP, PAA	Not reported	Increased CRP and PAA levels were associated with delirium occurrence
Plaschke (2007)	SAA, APTT, INR, CRP, bilirubin, glucose, K, Na, albumin, IL-6, urea, Hb	On the 2nd day following ICU admission	Venous blood, site unknown	Hb, urea	Not reported	Increased urea was associated with delirium occurrence Decreased haemoglobin was associated with delirium occurrence
Watts (2007)	WBC, neutrophils, Hb, CRP, lactate	On each day of ICU admission	*NS*	None		No significant finding
Seaman (2006)	Hb, haematocrit	“Routine clinical practice” Delirium: average of the two lowest values in 48 h before delirium onset Non-delirium: average of two lowest values in the 1st 48 h of admission	*NS*	Hb, haematocrit	Not reported	Decreased haemoglobin and haematocrit levels were associated with delirium occurrence

Schematic demonstration of the biomarkers investigated in each included study. Biomarkers are listed if the statistical analyses presented in the original studies were conducted to assess the association between biomarkers and delirium occurrence, -severity, -duration, or -subtype. Biomarkers demonstrating a statistically significant association are highlighted and reported in separate columns as either unadjusted or adjusted values, depending on the statistical approach applied in the original analysis. Main conclusions are presented according to the most detailed statistical analyses performed. Due to heterogeneity in methodology and outcome presentation, the main conclusions were based on the judgement of the review authors

*NS* not specified, *ICU* intensive care unit, *CAM-ICU* Confusion Assessment Method for the Intensive Care Unit

Biomarker abbreviations are not expanded in this table due to space limitations. Full definitions are provided in Appendix B

^*^The study reported an association with ‘persistent delirium’ (delirium present upon hospital discharge), a term introduced by the original study authors

#### Overall summary

Inflammatory, metabolic/endocrine, haematological, and neurobiological markers constituted the most extensively investigated biomarker categories. Despite substantial methodological variability, the most consistent associations were observed for inflammatory markers, selected neurobiological markers and leukocyte-derived haematological indices. Evidence from coagulation and endothelial markers, organ dysfunction indices, amino acid-related metabolites, and omics-based approaches was sparse and yielded heterogeneous findings. Across studies, pro-inflammatory cytokines [42, 59, 75, 80, 81] and acute-phase reactants [46, 53, 59, 66, 74, 75, 82, 93, 97], together with leukocyte-derived haematological indices [37, 39, 56, 60, 90], were repeatedly associated with delirium occurrence in adjusted analyses and, in some studies, with longer duration [42, 59, 70, 82, 93] and greater severity [46, 59]. Neurobiological injury markers were generally less frequently studied, although three markers were evaluated in multiple cohorts and showed predominantly positive associations across outcomes in adjusted analyses [37, 51, 59, 71, 72, 96]. The variability in biomarker sampling strategies precludes definitive conclusions regarding temporality. Overall, reproducible signals were concentrated within inflammatory and haematological domains, whereas other biological pathways remain insufficiently validated. Table 3 lists the biomarkers examined in each study, the timing and site of sample collection, and statistically significant associations with delirium outcomes in unadjusted and adjusted analyses, respectively. Supplementary Figure A–H (in Supplementary Materials 2) provides a visual overview of the direction and adjustment status of associations across studies.

#### Haematological markers

Fourteen haematological serum biomarkers were evaluated across 17 studies with 12 markers assessed in at least two studies. Seven biomarkers were white blood cell indices, five were red blood cell indices and two were platelet indices. Twelve markers demonstrated a significant association with delirium: seven in unadjusted analyses (white blood cell (WBC) count, monocytes, red blood cell (RBC) count, red cell distribution width (RDW), haemoglobin, haematocrit and platelets) and five in adjusted analyses (neutrophils, lymphocytes, neutrophil-to-lymphocyte ratio (NLR), lymphocyte-to-monocyte ratio (LMR) and platelet-to-lymphocyte ratio (PLR)). Detailed results are provided in Table 3 with a visual overview presented in Supplementary Figure A.

#### Coagulation and endothelial dysfunction

Nine serum biomarkers were examined across nine studies, with six assessed in at least two studies. Six biomarkers were related to the coagulation cascade and fibrinolysis and three to endothelial activation and injury. Five markers demonstrated a significant association with delirium: one in unadjusted analyses [international normalized ratio (INR)] and four in adjusted analyses (protein C, D-dimer, plasminogen activator inhibitor 1 (PAI-1) and E-selectin). Detailed results are provided in Table 3 with a visual overview presented in Supplementary Figure B.

#### Inflammatory, infectious and immunological markers

Forty-nine serum biomarkers (including 37 inflammatory, one infectious, and 11 immunological markers) were assessed across 33 studies, with 18 markers examined in at least two studies. Seven of the inflammatory biomarkers were acute-phase reactants, 17 were cytokines, six were chemokines, six were immune effector enzymes and granule proteins, and two were hematopoietic growth factors. Of the immunological biomarkers, six were related to immune cells and five to humoral immunity. Twenty-four markers demonstrated a significant association with delirium: 12 in unadjusted analyses (neopterin, serum galactin-3, interleukin (IL)-18, IL-1RA, C–C motif chemokine ligand (CCL) 2 and 3, C-X-C motif chemokine ligand (CXCL) 1 and 10, monocyte chemoattractant protein-1 (MCP-1), macrophage migration inhibitory factor (MIF), CD14^hi^CD16^−^ monocyte subtype and natural killer (NK) cells) and 12 in adjusted analyses (C-reactive protein (CRP), procalcitonin (PCT), ferritin, adiponectin, IL-1β, IL-6, IL-8, IL-10, tumour necrosis factor alpha (TNF-α), soluble tumour necrosis factor receptors (sTNFR) 1 and 2 and matrix metallopeptidase-9 (MMP-9)). Detailed results are provided in Table 3 with a visual overview presented in Supplementary Figure C.

#### Neurobiological markers

Twenty-six serum biomarkers were examined across 23 studies, with 11 markers assessed in at least two studies. Eight biomarkers were related to neuronal injury and structural damage, two to synaptic function and neuroplasticity, 12 to neurodegeneration and four to neurotransmission. Sixteen markers showed a significant association with delirium: 10 in unadjusted analyses (neurofilament heavy chain (NfH), glial fibrillary acidic protein (GFAP), neurogranin (Ng), total tau (t-tau), tau/amyloid beta (Aβ)_1–42_ ratio, Aβ_1–40_, Aβ_N-40_, Aβ_N-42/40_ ratio, plasma anticholinergic activity (PAA) and serum acetylcholinesterase (s-AchE) level) and six in adjusted analyses (S100 calcium-binding protein B (S-100β), neuron-specific enolase (NSE), neurofilament light chain (NfL), brain-derived neurotrophic factor (BDNF), ubiquitin carboxy-terminal hydrolase L1 (UCHL1) and Aβ_1–42/40_ ratio). Detailed results are provided in Table 3 with a visual overview presented in Supplementary Figure D.

#### Organ injury markers

Twelve serum biomarkers were examined across 20 studies, with six markers assessed in at least two studies. Five biomarkers were related to liver function, one to pancreatic function, one to muscle injury, one to renal function, one to unspecific tissue damage and three to cardiac function. Eight markers demonstrated a significant association with delirium: seven in unadjusted analyses (alanine aminotransferase (ALAT), aspartate aminotransferase (ASAT), gamma-glutamyl transferase (γ-GT), albumin, creatinine, troponin-I and NT-proBNP) and one in adjusted analyses (bilirubin). Detailed results are provided in Table 3 with a visual overview presented in Supplementary Figure E.

#### Metabolic and endocrine function

Twenty-three metabolic and four endocrine serum biomarkers were examined across 27 studies, with 13 assessed in at least two studies. Four of the metabolic biomarkers were related to renal metabolism and nitrogen balance, seven to glucose and lipid metabolism, five to acid–base balance and lactate metabolism, five to electrolyte balance and two were alcohol-related markers. Twenty-one markers demonstrated a significant association with delirium: eight in unadjusted analyses (insulin-like growth factor 1 (IGF-1), blood urea nitrogen (BUN), uric acid, urea, glucose, blood pH, bicarbonate and triglycerides) and 13 in adjusted analyses (leptin, serum cortisol, prolactin, blood urea creatinine ratio (BCR), lactate, lactate clearance rate, anion gap, sodium, potassium, triglyceride-glucose (TyG) index, triglyceride-glucose (TyG) average (AVG), carbohydrate-deficient transferrin (CDT) and Anttila index). Detailed results are provided in Table 3 with a visual overview presented in Supplementary Figure F.

#### Amino acids metabolism

Eight serum biomarkers were examined across two studies, with none being assessed in both. A list of biomarkers in the category is provided in Appendix B. Seven markers demonstrated a significant association with delirium: five in unadjusted analyses (ascorbic acid, kynurenic acid, kynurenine, kynurenine/tryptophan ratio, tryptophan) and two in adjusted analyses (tyrosine/large neutral amino acids (Tyr/LNAA) ratio, tryptophan/large neutral amino acids (Trp/LNAA) ratio). Detailed results are provided in Table 3 with a visual overview presented in Supplementary Figure G.

#### Omics-based exploratory biomarkers

Eight biomarkers were examined across three studies, with none assessed in at least two studies. Five markers were examined using proteomics, two markers using transcriptomics and one marker using spectromics. Eight markers showed a significant association with delirium: all were in unadjusted analyses and none in adjusted analyses. Detailed results are provided in Table 3 with a visual overview presented in Supplementary Figure H.

### Discriminative and diagnostic performance

Fourteen studies assessed the discriminative performance of the individual biomarkers [37, 41, 43, 52, 56, 64, 67, 68, 70, 74, 76, 83, 93, 96], including BDNF, NF-L, NSE, S100B, CRP, prolactin, lymphocyte and NK cell counts, CD14^hi^CD16^−^ monocyte percentage, NLR, serum galectin-3, leptin, cortisol, CDT, and the Anttila index. Most biomarkers demonstrated moderate discrimination (AUC 0.68–0.77) while higher performance was reported for NK cell count (AUC 0.895; sensitivity 80.2%; specificity 80.8%) [56], serum galectin-3 (AUC 0.87; sensitivity 92.9%; specificity 66.7%) [67], and S100B (AUC 0.856; sensitivity 88.2%; specificity 63.0%) [67].

Eight studies evaluated multivariable models combining biomarkers with clinical or demographic variables [39, 50, 70, 75, 76, 91, 93, 96] yielding modest to high discrimination (AUC 0.70–0.99). The strongest performance was observed for a model including seven spectral bands (AUC 0.99; sensitivity 92%; specificity 92%) [91] followed by a multivariable logistic regression model integrating admission cortisol levels, day 2 S100B, clinical covariates, and an ‘age x cortisol’ interaction (AUC 0.89; 95% CI 0.82–0.95) [76].

### Study quality

Most of the included studies were of poor quality based on the NOS converted to AHRQ standards. The mean total NOS score was seven among case–control studies and approximately six among cohorts. Twenty studies (33%) were categorized as ‘good quality’ [40, 42, 43, 57, 58, 60, 61, 63, 68, 69, 71–76, 80, 83, 84, 97], 10 (16%) were categorized as ‘fair quality’ [38, 39, 46, 47, 50, 55, 59, 70, 90, 92] and 30 (49%) were categorized as ‘poor quality’ [37, 41, 44, 46, 48, 49, 51–54, 62, 64–67, 77–79, 81, 82, 85–89, 91, 93–96]. One study (2%) included both a retrospective and prospective cohort categorized as ‘fair quality’ and ‘poor quality’, respectively [56]. There was substantial variation in quality across several domains. Details on individual studies and domain-specific ratings are provided in Supplementary Table 2 (in Supplementary Materials 1).

## Discussion

This systematic review identified 153 blood-based biomarkers across 61 studies involving 39,354 adult ICU patients. One-hundred-and-one biomarkers were significantly associated with delirium, of which 58 were significant in unadjusted analyses, and 43 in adjusted analyses. Only 40 biomarkers were statistically associated with delirium in at least two studies.

The present review focuses on blood-based biomarkers of delirium in adult ICU patients. Five existing systematic reviews [20–24] have investigated biomarkers of delirium, mostly in other patient populations [20–23], but knowledge remains sparse. Moreover, this review provides an overview of sampling methods and delirium assessment, and includes studies published up to 3rd September 2025.

### Mechanistic implications of biomarker associations

Across biomarker categories, the most reproducible signals were observed within inflammatory pathways, leukocyte-derived indices, and selected markers of neuronal injury. The predominance of associations within inflammatory and leukocyte-derived markers supports the conceptualization of delirium as a neuro-inflammatory condition [6]. The repeated positive associations observed for CRP [53, 59, 74, 93, 97], IL-6 [42, 59], IL-8 [42, 59, 81], TNF-α [42, 59], soluble TNF receptors (sTNFR1/2) [42, 75, 80], and related acute-phase reactants [46, 66, 75, 82] are consistent with a framework in which systemic immune activation contributes to blood–brain barrier dysfunction, microglial activation, and synaptic disturbance, culminating in acute brain failure [6]. Systemic inflammation may function both as a precipitating factor and an amplifying mechanism in acute brain failure, particularly among vulnerable critically ill patients. Nevertheless, these markers are also closely linked to overall illness severity and sepsis-related organ dysfunction [109], limiting their specificity. The predominance of single-timepoint sampling further constrains causal inference, and the lack of replication, inconsistent adjustment strategies, and substantial methodological heterogeneity preclude definitive conclusions regarding mechanistic primacy or specificity.

Neurobiological markers such as S100 calcium-binding protein B (S100β), neurofilament light chain (NfL), and neuron-specific enolase (NSE) were among the most frequently examined, and were predominantly positively associated with delirium in adjusted analyses [37, 51, 59, 71, 72, 96]. These proteins reflect astroglial activation [110], axonal injury and neuronal stress [111], suggesting that delirium in critical illness may involve structural or cellular injury in addition to transient functional disturbance [6]. However, whether these markers reflect delirium-specific mechanisms or broader neurological vulnerability remains uncertain, particularly as adjustment for baseline cognition, sedation depth, and primary brain injury was inconsistently performed. At the same time, their interpretation is complicated by the biological compartment in which they are measured. While cerebrospinal fluid may capture central nervous system pathology more accurately, the need for invasive sampling limits its applicability, rendering blood-based biomarkers as the most feasible option. Notably, certain markers such as NSE [37], NfL [112] and GFAP [113] have demonstrated robust correlations with CSF levels. Nevertheless, peripheral concentrations may be influenced by extra-cerebral factors, and given the incomplete understanding of delirium pathophysiology, it remains unclear whether the most informative biomarkers are confined to the central nervous system.

Leukocyte-derived ratios (e.g. NLR and LMR) provided additional support for a role of peripheral immune imbalance, although evidence remains limited to a small number of cohorts [39, 56, 90]. In contrast, biomarkers targeting neurotransmitter pathways, coagulation, endothelial dysfunction, metabolic dysregulation, and amino acid metabolism were investigated less frequently and yielded heterogeneous results, offering limited mechanistic coherence.

### The ideal biomarker

The clinical value of novel biomarkers remains limited unless they contribute meaningfully to improved treatment, inform changes in clinical practice, or support decision-making in ways that enhance patient outcomes. Although numerous candidate biomarkers have been investigated based on pathophysiological hypotheses, none have yet demonstrated sufficient diagnostic accuracy to warrant clinical use [20, 22]. Only a limited number of studies assessed diagnostic performance [37, 39, 41, 43, 50–52, 56, 67, 68, 70, 74–76, 83, 96], while the remaining focused on statistical associations with delirium. Adequate diagnostic performance requires considerations concerning sensitivity and specificity [114]. A highly sensitive but poorly specific biomarker may detect most cases, yet risk overdiagnosis and overtreatment by misclassifying non-delirious patients. Conversely, a highly specific but insensitive biomarker may confirm disease when present but fail to identify cases, leading to underdiagnosis and missed opportunities for care.

Moreover, any biomarker must yield interpretable and stable results across repeated measurements and remain valid across the heterogeneous ICU population, characterized by high multimorbidity and interacting physiological disturbances [115]. This complicates both the interpretation of biomarker signals and the attribution of changes to a single underlying cause [116]. While high sensitivity is often considered beneficial, it may paradoxically introduce uncertainty when applied to a condition as complex and heterogeneous as delirium. As a multifactorial syndrome with no clearly defined pathophysiology, delirium emerges from different interacting mechanisms that vary across patients and clinical contexts [9]. Highly sensitive biomarkers may detect subtle biological changes, but in the absence of causal clarity, they risk reflecting abnormalities of uncertain clinical relevance [19]. Hence, the search for one or few generalizable biomarkers might oversimplify a biologically diverse condition. Yet, the alternative of context-specific markers with narrow indications for use may reduce feasibility and exclude many patients from potential benefit. Given the acute and dynamic nature of the ICU setting, the clinical utility of biomarkers is tightly linked to feasibility. Timely availability is essential, as delay in results may lead to delayed care [117]. Biomarkers must be rapidly and easily obtained, require a minimum of processing, be cost-effective, and provide reliable, consistent results under variable preanalytical conditions [118]. Some of the evaluated biomarkers in this review require considerable time, effort and cost to measure, limiting their applicability in routine clinical care.

### Heterogeneity in delirium research

Methodological heterogeneity constrains the present review and reflects general challenges in delirium biomarker research.

One contributing factor is variability in study populations. Studying more homogeneous ICU cohorts, as proposed by Sobbi and Van den Boogaard [119], may reduce interaction between pathophysiological pathways. Yet, this would limit generalizability and restrict applicability to a narrow subgroup of ICU patients [116]. Another key limitation concerns the unknown optimal timing of sampling. The fluctuating nature of delirium hampers the alignment of single timepoint sampling with various phases of the syndrome and thus may fail to capture relevant changes. Many studies sampled within 24 h of ICU admission, despite evidence that some biomarkers peak later, increasing their risk of missed associations. Although previous literature [22, 71, 119, 120] has suggested serial measurements, less than half of the studies applied longitudinal sampling (see Table 3 for references). Serial measurements could allow assessment of biomarker levels prior to onset, tracking of their trajectory, and determination of whether they resolve in parallel with clinical recovery. Moreover, no consensus exists on optimal sampling intervals, and lack of alignment with delirium assessments further complicates interpretation. 

Variation in delirium assessment, follow-up periods and reported outcomes represent further limitations. Most studies relied on infrequent assessments, thereby risking missed transient episodes and potential misclassification of patients with a single negative screening as ‘delirium-free’ for the entire day. Such misclassification could erroneously miss true associations. In addition, insufficient reporting and inconsistencies in follow-up duration complicate interpretation. Given the variable timing of delirium onset, short or unspecified follow-up periods or follow-up limited to the duration of the ICU stay may result in missed cases if delirium develops after ICU discharge yet remains related to the critical illness. This increases the risk of outcome misclassification, as samples may have been collected from patients incorrectly classified as delirium-free, because delirium manifested later. This may lead to misinterpretation of the association, and the biomarker may be mistakenly considered indicative of a non-delirium state. Furthermore, variability in reported delirium outcomes across studies further hampers comparability and underscores the need for adherence to standardized outcome definitions [121]. Ultimately, the methodological differences highlighted above emphasize the need for improved standardization throughout the field.

### Strengths and limitations

Strengths of this review include adherence to the PRISMA guideline [26] and a comprehensive, systematic literature search. The search strategy was reviewed by an information specialist from a university medical library to ensure quality and reduce the risk of missing relevant literature. Moreover, the included studies were critically appraised and study quality was systematically assessed by applying the NOS and AHRQ standards. To our knowledge, this is also the first systematic review to provide a structured and extensive overview of biomarker sampling and delirium assessment within ICU studies evaluating biomarkers in relation to delirium in critically ill patients.

We acknowledge that this review also has a number of limitations. First, the methods of the majority of the included studies were of variable quality. Most studies did not demonstrate absence of the outcome at baseline and lacked information concerning loss to follow-up, completeness of the data, or handling of missing data. Furthermore, many studies failed to adjust for or match on predefined confounders considered essential for comparability. However, distinguishing between confounders and effect mediators is inherently difficult—a challenge that is further amplified by the complexity of ICU pathophysiology and the limited understanding of the underlying mechanisms of delirium. As a result, statistical adjustments may inadvertently attenuate true associations if variables on causal pathways are mistakenly treated as confounders [122]. Moreover, many studies employed single-cohort designs with small sample sizes (see references in Table 1), which most likely underpowered the ability to detect true associations [123] and limited the ability to adjust for multiple confounders due to the risk of overadjustment [124]. Second, given the observational design of all included studies, a substantial risk of bias remains due to residual unknown confounding. Third, this review identified significant associations for approximately 66% of the examined biomarkers. This high rate may reflect publication bias, as studies reporting no associations may be underrepresented in the published literature. Fourth, although the literature search was systematic and the search strategy was reviewed by an information specialist, the heterogeneous and inconsistent terminology in delirium research [125, 126] impeded the database search and may have led to omission of relevant studies, despite broad MeSH terms aimed to capture all studies on delirium. Finally, our synthesis was limited to qualitative conclusions due to the substantial methodological heterogeneity, which precluded meta-analysis. The strengths of identified associations could not be reliably quantified without risking a misleading average, given the variability in study populations, analytical approaches and methodological differences in the timing of biomarker sampling, delirium assessment frequency and follow-up duration.

### Future perspectives

Future advancement depends on methodological alignment across studies including unified terminology, standardized sampling frameworks, and consistent delirium assessment. International academic consensus is needed to reduce variability and enable meaningful replication. Multicohort studies may improve statistical power and generalizability, combining biomarkers in panels and aiding interpretation through AI models may enhance signal detection. However, future clinical utility will ultimately depend on the availability of rapid assays and diagnostic precision.

## Conclusion

In summary, 153 biomarkers have been investigated, of which 101 were statistically significant and 40 were replicated across at least two studies. Reproducible signals were largely confined to inflammatory, haematological and neurobiological domains while other pathways lacked consistent replication. Many biomarkers are potentially interesting, although clinical implementation remains a distant prospect and all must undergo further rigorous development, including analytical and clinical validation, and demonstration of clinical value and feasibility. This review underlines obstacles in delirium biomarker research, particularly the substantial heterogeneity across studies, with most being underpowered and characterized by poor methodological quality and critical statistical limitations. Future studies adhering to standardized methodology are needed to improve comparability and establish firm conclusions.

## Take-home message

By organizing the vast and complex evidence base on blood-based biomarkers and ICU-acquired delirium, we aim to provide a valuable comprehensive resource to guide future mechanistic and interventional research on ICU-acquired delirium. Reproducible signals were largely confined to inflammatory, haematological and neurobiological domains while other pathways lacked consistent replication. Future studies adhering to standardized methodology are needed to improve comparability and establish firm conclusions.

## Supplementary Information

Supplementary material 1.

Supplementary material 2.

## Data Availability

The data used for the synthesis in this study are available from the corresponding author upon reasonable request. Study quality assessment details are also available upon reasonable request.
