# Supplementary material for: Blood-based biomarkers and delirium in critically ill adults in the ICU: a systematic review
Source: Intensive Care Med Exp. 2026 Apr 20;14:48. doi: 10.1186/s40635-026-00894-5 (PMC13096256; doi:10.1186/s40635-026-00894-5)
Supplement: Supplementary file 1 — Supplementary material 1. [file 40635_2026_894_MOESM1_ESM.docx]

***Supplementary materials***

**Blood-based biomarkers and delirium in critically ill adults in the ICU:**

**A systematic review**

**Journal:**

Intensive Care Medicine Experimental

**Authors:**

Julie Kathrine Ryberg Sankel^1^, Cand. Med.

Naia Bech Cranø^1^, Cand. Med.

Pär Ingemar Johansson^2, 3^, Professor, M.D., D.M.Sc., MPA

Lars Peter Kloster Andersen^1, 3^, Ass. Professor, M.D, Ph.D.

**Corresponding author:**

Julie Kathrine Ryberg Sankel. Centre for Anaesthesiological Research, Department of Anaesthesiology, Zealand University Hospital, Lykkebækvej 1, 4600 Køge, Denmark.

E-mail: juliesankel@gmail.com. Phone: +45 50 55 05 28.

**Table of contents:**

- Appendix A. Search protocol
- Appendix B. Biomarker categories
- Supplementary Table 1. Patient characteristics and additional outcomes
- Supplementary Table 2. Study quality assessments

**Appendix A. Search protocol**

This appendix provides the search protocol used in the review.

| **Search Date** | **Database** | **Search strategy** | **Search results / no. of hits** |
| --- | --- | --- | --- |
| September 3, 2025 | PubMed  National Library of Medicine,  *1946 -* | **#1**: (((((((delirium[MeSH Terms]) OR (delirium[Text Word])) OR ("acute brain dysfunction"[Text Word])) OR ("acute brain failure"[Text Word]) OR ("ICU psychosis"[Text Word])) OR (encephalopathy[Text Word])) OR ("acute confusional state"[Text Word])) OR ("acute confusion"[Text Word]))  **#2**: ((((((critical illness[MeSH Terms]) OR (critical* ill*[Text Word])) OR (ICU[Text Word])) OR ("intensive care unit"[Text Word])) OR ("critical care"[Text Word])) OR ("critical care unit"[Text Word])) OR ("acute* ill*"[Text Word])  **#3**: ((((((((Biomarkers [MeSH Terms]) OR ("blood biomarker*"[Text Word])) OR ("plasma biomarker*"[Text Word])) OR ("biological marker*"[Text Word])) OR ("clinical marker*"[Text Word])) OR ("serological marker*"[Text Word])) OR (delirium[Text Word] AND profile*[Text Word]) ) OR (biomarker*[Text Word]))  **#4**: #1 AND #2 AND #3 | [96,927](https://pubmed.ncbi.nlm.nih.gov/?term=%28%28%28%28%28%28%28delirium%5BMeSH+Terms%5D%29+OR+%28delirium%5BText+Word%5D%29%29+OR+%28%22acute+brain+dysfunction%22%5BText+Word%5D%29%29+OR+%28%22acute+brain+failure%22%5BText+Word%5D%29+OR+%28%22ICU+psychosis%22%5BText+Word%5D%29%29+OR+%28encephalopathy%5BText+Word%5D%29%29+OR+%28%22acute+confusional+state%22%5BText+Word%5D%29%29+OR+%28%22acute+confusion%22%5BText+Word%5D%29%29&sort=date)  [317,523](https://pubmed.ncbi.nlm.nih.gov/?term=%28%28%28%28%28%28critical+illness%5BMeSH+Terms%5D%29+OR+%28critical%2A+ill%2A%5BText+Word%5D%29%29+OR+%28ICU%5BText+Word%5D%29%29+OR+%28%22intensive+care+unit%22%5BText+Word%5D%29%29+OR+%28%22critical+care%22%5BText+Word%5D%29%29+OR+%28%22critical+care+unit%22%5BText+Word%5D%29%29+OR+%28%22acute%2A+ill%2A%22%5BText+Word%5D%29&sort=&size=100)  [1,269,287](https://pubmed.ncbi.nlm.nih.gov/?term=%28%28%28%28%28%28%28%28Biomarkers+%5BMeSH+Terms%5D%29+OR+%28%22blood+biomarker%2A%22%5BText+Word%5D%29%29+OR+%28%22plasma+biomarker%2A%22%5BText+Word%5D%29%29+OR+%28%22biological+marker%2A%22%5BText+Word%5D%29%29+OR+%28%22clinical+marker%2A%22%5BText+Word%5D%29%29+OR+%28%22serological+marker%2A%22%5BText+Word%5D%29%29+OR+%28delirium%5BText+Word%5D+AND+profile%2A%5BText+Word%5D%29+%29+OR+%28biomarker%2A%5BText+Word%5D%29%29&sort=&size=100)  [**456**](https://pubmed.ncbi.nlm.nih.gov/?term=%238+AND+%239+AND+%2310&sort=date) |
| September 3, 2025 | Embase  Ovid,  *1974 -* | **#1**: exp delirium/  **#2**: (delirium or "acute brain failure" or "acute brain dysfunction" or "ICU psychosis" or encephalopathy or "acute confusional state" or "acute confusion").mp. [mp=title, abstract, heading word, drug trade name, original title, device manufacturer, drug manufacturer, device trade name, keyword heading word, floating subheading word, candidate term word]  **#3**: #1 OR #2  **#4**: exp critical illness/  **#5**: ("critical* ill*" or ICU or "intensive care unit" or "critical care" or "critical care unit" or "acute* ill*").mp. [mp=title, abstract, heading word, drug trade name, original title, device manufacturer, drug manufacturer, device trade name, keyword heading word, floating subheading word, candidate term word]  **#6**: #4 OR #5  **#7**: exp biological marker/  **#8**: ("blood biomarker*" or "plasma biomarker*" or "biological marker*" or "clinical marker*" or "serological marker*" or (delirium and profile*) or biomarker*).mp. [mp=title, abstract, heading word, drug trade name, original title, device manufacturer, drug manufacturer, device trade name, keyword heading word, floating subheading word, candidate term word]  **#9**: #7 OR #8  **#10**: #3 AND #6 AND #9 | 51,075  179,728  180,347  40,792  601,225  601,225  578,884  965,489  965,489  **1099** |
| September 3, 2025 | The Cochrane Library  *1992 -* | **#1**: MeSH descriptor: [Delirium] explode all trees  **#2**: (delirium OR "acute brain failure" OR "acute brain dysfunction" OR "ICU psychosis" OR encephalopathy OR "acute confusional state" OR "acute confusion"):ti,ab,kw  **#3**: #1 OR #2  **#4**: MeSH descriptor: [Critical Illness] explode all trees  **#5**: ("critical illness" OR "critically ill" OR ICU OR "intensive care unit" OR "critical care" OR "critical care unit" OR "acute illness" OR "acutely ill"):ti,ab,kw  **#6**: #4 OR #5  **#7**: MeSH descriptor: [Biomarkers] explode all trees  **#8**: (blood NEXT biomarker or plasma NEXT biomarker* or biological NEXT biomarker* or clinical NEXT marker* or serological NEXT marker* or (delirium AND profiles) or biomarker*):ti,ab,kw  **#9**: #7 OR #8  **#10**: #3 AND #6 AND #9 | 1,712  11,316  11,316  3,807  43,454  44,545  31,688  61,641  68,273  **77** |
| September 3, 2025 | Web of Science  Clarivate, *1900-* | **#1**: TS=(delirium OR "acute brain failure" OR "acute brain dysfunction" OR "ICU psychosis" OR encephalopathy OR "acute confusional state" OR "acute confusion")  **#2**: TS=("critical illness" OR "critically ill" OR ICU OR "intensive care unit" OR "critical care" OR "critical care unit" OR "acute illness" OR "acutely ill")  **#3**: (((((((TS=(Biomarkers)) OR ALL=("blood biomarker*")) OR ALL=("plasma biomarker*")) OR ALL=("biological marker*")) OR ALL=("clinical marker*")) OR ALL=("serological marker*")) OR ALL=( delirium AND profile*)) OR ALL=( biomarker*)  **#4**: #1 AND #2 AND #3 | 116,166  330,059  692,573  **393** |

**Appendix B. Biomarker categories**

This appendix presents the categorization of biomarkers and lists the specific biomarkers included in each category.

**1. Haematological markers**

*White blood cell indices*: white blood cell count (WBC), leukocytes, neutrophils, lymphocytes, monocytes, lymphocyte-to-monocyte ratio (LMR), neutrophil-to-lymphocyte ratio (NLR).

*Red blood cell indices*: red blood cell count (RBC), red cell distribution width (RDW), erythrocyte sedimentation rate (ESR), haemoglobin (Hb), haematocrit (Hct).

*Platelet indices*: platelets, platelet-to-lymphocyte ratio (PLR).

**2. Coagulation and endothelial dysfunction**

*Coagulation cascade and fibrinolysis*: Protein C, international normalized ratio (INR), prothrombin time (PT), activated partial thromboplastin time (PTT), D-dimer, plasminogen activator inhibitor-1 (PAI-1).

*Endothelial activation and injury*: E-selectin, angiopoietin-2 (Ang-2), von Willebrand factor antigen (vWF antigen).

**3. Inflammatory, infectious and immunological markers**

*Acute phase reactants*: C-reactive protein (CRP), procalcitonin (PCT), ferritin, neopterin, adiponectin, serum galectin-3, serum apolipoprotein E (s-ApoE).

*Cytokines*: interleukin (IL)-1α, IL-1β, IL-2, IL-4, IL-5, IL-6, IL-8, IL-10, IL-12, IL-17A, IL-18, IL-1 receptor antagonist (IL-1RA), interferon gamma (IFN-γ), tumor necrosis factor alpha (TNF-α), soluble tumor necrosis factor receptors (sTNFR) 1 and 2.

*Chemokines*: C-C motif chemokine ligand (CCL) 2 and 3, C-X-C motif chemokine ligand (CXCL) 1 and 10, macrophage inflammatory protein-1β (MIP-1β), monocyte chemoattractant protein-1 (MCP-1).

*Immune effector enzymes and granule proteins:* matrix metallopeptidase-9 (MMP-9), myeloperoxidase (MPO), macrophage migration inhibitory factor (MIF), neutrophil gelatinase-associated lipocalin (NGAL), substance P (SUB P), human neutrophil peptide 1 (HNP-1).

*Haematopoietic growth factors*: granulocyte-colony stimulating factor (G-CSF), granulocyte-macrophage colony-stimulating factor (GM-CSF).

*Infectious biomarker*: (1,3)-β-D-glucan (BDG)

*Humoral immunity*: immunoglobulin A (IgA), IgG, IgM, complement component C3 and C4.

*Immune cells:* natural killer (NK) cells, B cells, T cells, CD14^hi^CD16⁻ monocytes, CD14^lo^CD16⁺ monocytes, CD14^hi^CD16⁺ monocytes.

**4. Neurobiological markers**

*Neuronal injury and structural damage*: S100 calcium-binding protein B (S-100β), neuron-specific enolase (NSE), neurofilament light chain (NfL), neurofilament heavy chain (NfH), BCL2/adenovirusE1B19kDa interacting protein 3 L (BNIP3L), ubiquitin carboxy-terminal hydrolase L1 (UCHL1), glial fibrillary acidic protein (GFAP), chitinase-3-like protein 1 (CHI3L1).

*Synaptic function and neuroplasticity*: neurogranin (Ng), brain-derived neurotrophic factor (BDNF).

*Neurodegeneration*: total tau (t-tau), tau/Aβ_1–42_ ratio, amyloid-β_1–42_ (Aβ_1–42_), amyloid-β_1–40_ (Aβ_1–40_), Aβ_1–40/42_ ratio, Aβ_1–42/40_ ratio, Aβ_N-42_, Aβ_N-40_, Aβ_N-42/40_ ratio, Aβ_1-42/N-42_ ratio, Aβ_1-40/N-40_ ratio, Triggering Receptor Expressed on Myeloid Cells 2 (TREM2)

*Neurotransmission*: serotonin, plasma anticholinergic activity (PAA), serum anticholinergic activity (SAA), serum acetylcholinesterase (s-AchE) level

**5. Organ injury markers**

*Liver function*: alanine aminotransferase (ALAT), aspartate aminotransferase (ASAT), gamma-glutamyl transferase (γ-GT), albumin, bilirubin.

*Pancreatic function*: amylase.

*Kidney function*: creatinine.

*Muscle injury*: creatine kinase (CK).

*Unspecific tissue damage*: lactate dehydrogenase (LDH).

*Cardiac function*: Creatine kinase MB isoenzyme (MB-CK), N-terminal pro-B-type natriuretic peptide (NT-proBNP), troponin-I.

**6. Metabolic and endocrine function**

*Renal metabolism and nitrogen balance*: blood urea nitrogen (BUN), blood urea creatinine ratio (BCR), urea, uric acid.

*Glucose and lipid metabolism*: glucose, triglycerides, triglyceride-glucose (TyG) index, triglyceride-glucose-average (TyG-AVG), total cholesterol, low-density lipoprotein (LDL), high-density lipoprotein (HDL).

*Alcohol-related biomarkers*: carbohydrate-deficient transferrin (CDT), Anttila index.

*Acid-base balance and lactate metabolism*: lactate, lactate clearance rate, blood pH, anion gap, bicarbonate.

*Electrolyte balance*: sodium (Na), potassium (K), calcium (Ca), chloride (Cl), phosphate.

*Endocrine function:* Insulin-like growth factor 1 (IGF-1), leptin, cortisol, prolactin.

**7. Amino acid metabolism**

Phenylalanine/large neutral amino acids (Phe/LNAA) ratio, tyrosine/large neutral amino acids (Tyr/LNAA) ratio, tryptophan/large neutral amino acids (Trp/LNAA) ratio, kynurenic acid, kynurenine, kynurenine/tryptophan ratio, tryptophan.

**8. Omics based exploratory markers**

*Proteomics*: paraoxonase 1 (PON1), thrombospondin 1 (THBS1), fibrinogen gamma chain (FGG), complement component 1q subcomponent C (C1QC), immunoglobulin heavy variable 3 (IgHV3).

*Transcriptomics*: period circadian regulator 2 mRNA (PER2 mRNA), heme oxygenase 1 mRNA (HO1 mRNA).

*Spectromics*: serum spectral bands.

**Supplementary Table 1. Patient characteristics and additional outcomes**

Patient characteristics and additional outcomes investigated for association to delirium other than biomarkers presented in the studies. An empty field represents that the given characteristic or outcome is not reported.

| First author (year) | Patient characteristics and additional outcomes | | | | | | | Additional examined parameters |
| --- | --- | --- | --- | --- | --- | --- | --- | --- |
|  | *Type of ICU / admission diagnoses* | *Comorbidities* | *ICU treatments and medications* | *Length of stay (hospital-, ICU- or both)* | *Disease severity scores* | *Recovery outcomes* | *Mortality* |  |
| Pham (2025) | - | - | X | X | X | X | X | - |
| Fayssoil (2025) | X | X | X | X | - | X | X | - |
| Zhang (2025) | - | N/A^c^ | N/A^c^ | N/A^c^ | N/A^c^ | - | - | - |
| Wang (2025) | - | X | X | X | X | - | X | Mendelian randomization of inflammatory blood cell ratios |
| Viegas (2024) | X | X | X | X | - | - | X |  |
| Zhang (2024) | - | X | - | X | X | - | X | Optic nerve sheath diameter + cerebrospinal fluid biomarkers |
| Torbic (2024) | N/A^b, c^ | N/A^b, c^ | N/A^b, c^ | - | N/A^b, c^ | - | N/A^b, c^ | - |
| Shi (2024) | - | X | X | - | X | - | - | - |
| Qian (2024) | - | X | X | - | X | - | X | - |
| Dragoescu (2024) | X | - | - | X | X | - | X | - |
| Brummel (2024) | N/A^a^ | N/A^a^ | N/A^a^ | N/A^a^ | N/A^a^ | - | N/A^a^ | - |
| Schreiber (2023) | N/A^a^ | - | - | X | X | - | X | - |
| Plaschke (2023) | - | - | X | X | X | - | X | - |
| Khan (2023) (A) | X^b^ | - | X^b^ | X^b^ | X^b^ | X^b^ | X^b^ | - |
| Khan (2023) (B) | - | N/A^a^ | N/A^a^ | - | N/A^a^ | - | N/A^a^ | - |
| Huang (2023) | - | X | X | X | - | - | X | - |
| Smith (2022) | X | X | X | X | X | - | X | - |
| Smeele (2022) | - | - | N/A^a^ | N/A^a^ | N/A^a^ | - | N/A^a^ | - |
| Park (2022) | X | X | X | X | X | - | - | - |
| Page (2022) | N/A^a^ | - | - | N/A^a^ | N/A^a^ | N/A^a^ | N/A^a^ | - |
| Lei (2022) | - | X | X | X | X | - | X | Transcriptomics of peripheral blood mononuclear cells |
| Seo (2021) | X | - | - | X | X | - | - | - |
| Steimer (2021) | - | N/A^c^ | - | N/A^c^ | N/A^c^ | - | N/A^c^ | - |
| Pektezel (2021) | X | X | X | X | X | - | X | - |
| Li (2021) | - | X | X | X | X | - | X | - |
| Souza-Dantas (2020) | N/A^a^ | N/A^a^ | N/A^a^ | N/A^a^ | N/A^a^ | - | N/A^a^ | Acute brain dysfunction phenotypes |
| Wanderlind (2020) | X | - | X | X | X | - | - | - |
| Voils (2020) | X | - | X | X | X | - | X | - |
| Khan (2020) | X^b^ | - | X^b^ | X^b^ | X^b^ | X^b^ | X^b^ | - |
| Jiang (2020) | X | X | X | X | X | - | - | - |
| Hayhurst (2020) | N/A^a^ | N/A^a^ | N/A^a^ | N/A^a^ | N/A^a^ | - | N/A^a^ | - |
| Cooper (2020) | - | N/A^c^ | N/A^c^ | - | N/A^c^ | - | - | - |
| Ozkul (2019) | - | - | - | - | - | - | - | - |
| Erikson (2019) | - | - | X | X | X | X | X | - |
| Ehler (2019) | N/A^d^ | - | N/A^c^ | N/A^c^ | N/A^d^ | N/A^d^ | N/A^c^ | Electroencephalography, Magnetic Resonance Imaging and cerebrospinal fluid biomarkers |
| Simons (2018) | X | X | X | X | X | - | X | - |
| Zhu (2017) | X | X | X | - | X | - | - | - |
| Li (2017) | X | X | X | X | X | - | X | - |
| Hemauer (2017) | N/A^a^ | - | N/A^a^ | N/A^a^ | N/A^a^ | - | - | - |
| Nguyen (2016) | N/A^a^ | N/A^a^ | X | X | N/A^a^ | - | X | - |
| Hughes (2016) | N/A^a^ | - | N/A^a^ | N/A^a^ | N/A^a^ | - | N/A^a^ | Peripheral artery tonometry |
| Anderson (2016) | N/A^c^ | N/A^c^ | N/A^c^ | - | N/A^c^ | - | N/A^c^ | - |
| Tomasi (2015) | X | - | X | X | X | - | X | - |
| Zhang (2014) | X | - | X | X | X | - | X | - |
| Ritter (2014) | X | - | X | - | X | - | X | - |
| Nguyen (2014) | N/A^a^ | N/A^a^ | X | - | X | - | X | - |
| Alexander (2014) | X | - | X | X | X | X | X | APOE genotype |
| Khan (2013) | N/A^b, c^ | - | N/A^b, c^ | N/A^b, c^ | N/A^b, c^ | - | N/A^b, c^ | - |
| Skrobik (2013) | - | - | - | N/A^a^ | X | - | - | Metabolism-related genetic polymorphisms |
| Sharma (2012) | X | X | X | X | X | - | X | - |
| Girard (2012) | N/A^a^ | - | - | - | N/A^a^ | - | N/A^a^ | - |
| Van den Boogaard (2011) | X | - | X | - | X | N/A^c^ | - | - |
| McGrane (2011) | N/A^a^ | - | N/A^a^ | N/A^a^ | N/A^a^ | - | N/A^a^ | - |
| Grandi (2011) | X | - | X | - | X | - | - | - |
| Uguz (2010) | - | X | X | X | - | - | X | - |
| Pandharipande (2009) | N/A^a^ | - | X | - | N/A^a^ | - | - | - |
| Pfister (2008) | N/A^d^ | - | N/A^d^ | - | N/A^d^ | - | N/A^d^ | Cerebral perfusion assessed with transcranial Doppler and near-infrared spectroscopy |
| Tsuruta (2008) | X | - | X | N/A^c^ | X | - | N/A^c^ | - |
| Plaschke (2007) | X | - | - | X | X | - | X | Electroencephalography |
| Watts (2007) | - | - | X | - | - | - | X | - |
| Seaman (2006) | X | - | - | - | X | - | - | - |

*N/A = not applicable.*

^a^ Outcome only reported on the total study cohort including both delirious and non-delirious patients.

^b^ Additional outcomes reported on the total study cohort. All included patients were delirious. No comparison with non-delirious patients.

^c^ Additional outcomes reported without examination of the association to delirium.

^d^ Additional outcomes reported individually on all included patients. No summary and descriptive statistical measures associating additional outcomes to delirium.

**Supplementary table 2. Study quality assessments**

Newcastle-Ottawa Scale (NOS) assessments and criteria for converting the total score to Agency for Health Research and Quality (AHRQ) standards.

***A) Criteria for conversion of the total NOS score to AHRQ standards***

- **Good quality**: 3-4 stars in selection domain AND 1-2 stars in comparability domain AND 2-3 stars in outcome/exposure domain
- **Fair quality**: 2 stars in selection domain AND 1-2 stars in comparability domain AND 2-3 stars in outcome/exposure domain
- **Poor quality**: 0-1 star in selection domain OR 0 stars in comparability OR 0-1 star in outcome/exposure domain

***B) Adapted NOS criteria for cohort study design***

For **cohort studies**: a star (★) is achieved in each domain if following criteria are met:

#1: Representativeness of patients with delirium (representative or somewhat representative)

#2: Representativeness of the comparable patients without delirium

#3: Ascertainment of biomarker status (medical record or laboratory tests)

#4: Ascertainment that delirium was not present at the start of study

#5: The study controls for age

#6: The study controls for severity of illness (APACHE or SOFA score) AND neurological status (sedation or coma)

#7: Assessment of delirium (independent assessment stated in the paper, or confirmation of delirium by reference to validated clinical assessment tool)

#8: Follow-up period sufficient for delirium to occur (defined as daily assessments during entire ICU stay described in the text)

#9: Adequacy of follow-up period data (complete follow up, low number of lost-to-follow-up (defined as < 20%), or descriptions provided of those lost to follow-up)

| **COHORT STUDIES** | | | | | | | | | | | |
| --- | --- | --- | --- | --- | --- | --- | --- | --- | --- | --- | --- |
| **First author**  **(year)** | **Selection** | | | | **Comparability** | | **Outcome** | | | **Total score** | **Overall quality**  *(AHRQ standards)* |
|  | *#1* | *#2* | *#3* | *#4* | *#5* | *#6* | *#7* | *#8* | *#9* |  |  |
|  | *Represen-tative cohort* | *Representative patients for comparison* | *Ascertainment of biomarker status* | *Demonstration that outcome of interest was not present at study initiation* | *Study controls for age* | *Study controls for severity of illness and neurological status* | *Outcome assessment* | *Sufficient follow-up period* | *Adequacy of follow-up data* |  |  |
| Pham (2025) | ★ | ★ | ★ |  | ★ | ★ | ★ |  |  | 6 | Poor |
| Fayssoil (2025) | ★ | ★ | ★ |  | ★ |  | ★ | ★ | ★ | 7 | Good |
| Zhang (2025) |  | ★ | ★ |  | ★ | ★ | ★ |  |  | 5 | Poor |
| Wang (2025) |  | ★ | ★ |  | ★ | ★ | ★ |  | ★ | 6 | Fair |
| Viegas (2024) | ★ | ★ | ★ | ★ |  |  | ★ |  |  | 5 | Poor |
| Zhang (2024) | ★ | ★ | ★ |  | ★ |  | ★ |  |  | 5 | Poor |
| Torbic (2024) | ★ |  | ★ |  | ★ |  | ★ |  | ★ | 5 | Fair |
| Shi (2024) |  | ★ | ★ |  |  | ★ | ★ |  | ★ | 5 | Fair |
| Qian (2024) |  | ★ | ★ | ★ | ★ | ★ | ★ | ★ | ★ | 8 | Good |
| Dragoescu (2024) |  | ★ | ★ |  |  |  | ★ | ★ | ★ | 5 | Poor |
| Brummel (2024) | ★ | ★ | ★ |  | ★ | ★ | ★ | ★ |  | 7 | Good |
| Schreiber (2023) | ★ | ★ | ★ |  | ★ |  | ★ |  | ★ | 6 | Good |
| Plaschke (2023) | ★ | ★ | ★ |  |  |  | ★ |  | ★ | 5 | Poor |
| Khan (2023) (A) |  |  | ★ |  |  |  | ★ | ★ | ★ | 4 | Poor |
| Khan (2023) (B) |  | ★ | ★ |  | ★ |  | ★ |  | ★ | 5 | Fair |
| Huang (2023) |  | ★ | ★ |  | ★ |  | ★ |  | ★ | 5 | Fair |
| Smith (2022) | ★ | ★ | ★ | ★ |  |  | ★ | ★ | ★ | 7 | Poor |
| Smeele (2022) |  | ★ | ★ |  |  |  | ★ |  |  | 3 | Poor |
| Park (2022) |  | ★ | ★ |  | ★ | ★ | ★ | ★ |  | 6 | Fair |
| Page (2022) |  | ★ | ★ |  |  |  | ★ | ★ |  | 4 | Poor |
| Lei (2022) | ★ | ★ | ★ |  |  |  | ★ |  |  | 4 | Poor |
| Seo (2021) |  | ★ | ★ | ★ |  |  | ★ | ★ |  | 5 | Poor |
| Steimer (2021) |  | ★ | ★ |  |  |  | ★ |  | ★ | 4 | Poor |
| Pektezel (2021) |  | ★ | ★ |  | ★ |  | ★ | ★ |  | 5 | Fair |
| Li (2021)  *1: Retrospective*  *2: Prospective* | ★  ★ | ★  ★ | ★  ★ |  | ★ |  | ★  ★ |  | ★  ★ | 6  5 | Fair  Poor |
| Souza-Dantas (2020) | ★ | ★ | ★ |  |  |  | ★ |  |  | 4 | Poor |
| Khan (2020) | ★ |  | ★ |  | ★ |  | ★ | ★ | ★ | 6 | Fair |
| Jiang (2020) | ★ | ★ | ★ |  | ★ |  | ★ | ★ | ★ | 7 | Good |
| Hayhurst (2020) | ★ | ★ | ★ |  | ★ |  | ★ | ★ | ★ | 7 | Good |
| Cooper (2020) |  | ★ | ★ |  |  |  | ★ |  | ★ | 4 | Poor |
| Erikson (2019) | ★ | ★ | ★ |  |  |  | ★ |  |  | 4 | Poor |
| Ehler (2019) | ★ | ★ | ★ |  |  |  | ★ |  | ★ | 5 | Poor |
| Simons (2018) |  | ★ | ★ | ★ | ★ |  | ★ |  |  | 5 | Poor |
| Zhu (2017) |  | ★ | ★ |  |  |  | ★ |  | ★ | 4 | Poor |
| Li (2017) |  | ★ | ★ | ★ | ★ | ★ | ★ | ★ | ★ | 8 | Good |
| Hemauer (2017) | ★ | ★ | ★ |  | ★ |  | ★ | ★ | ★ | 7 | Good |
| Nguyen (2016) |  | ★ | ★ |  | ★ | ★ | ★ | ★ |  | 6 | Fair |
| Hughes (2016) | ★ | ★ | ★ |  | ★ |  | ★ | ★ | ★ | 7 | Good |
| Anderson (2016) | ★ | ★ | ★ |  | ★ | ★ | ★ | ★ | ★ | 8 | Good |
| Tomasi (2015) | ★ | ★ | ★ | ★ |  | ★ | ★ | ★ | ★ | 8 | Good |
| Zhang (2014) | ★ | ★ | ★ | ★ | ★ |  | ★ | ★ |  | 7 | Good |
| Ritter (2014) | ★ | ★ | ★ | ★ | ★ | ★ | ★ |  | ★ | 8 | Good |
| Nguyen (2014) | ★ | ★ | ★ |  | ★ | ★ | ★ | ★ | ★ | 8 | Good |
| Alexander (2014) |  | ★ | ★ |  | ★ | ★ | ★ |  |  | 5 | Poor |
| Khan (2013) |  |  | ★ |  | ★ |  | ★ | ★ |  | 4 | Poor |
| Skrobik (2013) | ★ | ★ | ★ |  |  |  | ★ |  | ★ | 5 | Poor |
| Sharma (2012) | ★ | ★ | ★ |  |  |  | ★ | ★ | ★ | 6 | Poor |
| Girard (2012) | ★ | ★ | ★ |  | ★ |  | ★ | ★ | ★ | 7 | Good |
| Van den Boogaard (2011) | ★ | ★ | ★ | ★ |  |  | ★ | ★ | ★ | 7 | Poor |
| McGrane (2011) | ★ | ★ | ★ |  | ★ |  | ★ |  |  | 5 | Poor |
| Uguz (2010) |  | ★ | ★ |  | ★ |  | ★ | ★ | ★ | 6 | Fair |
| Pandharipande (2009) | ★ | ★ | ★ |  | ★ | ★ | ★ |  | ★ | 7 | Good |
| Pfister (2008) | ★ | ★ | ★ |  |  |  | ★ |  |  | 4 | Poor |
| Tsuruta (2008) | ★ | ★ | ★ |  |  |  | ★ |  |  | 4 | Poor |
| Plaschke (2007) | ★ | ★ | ★ |  |  |  | ★ |  | ★ | 5 | Poor |
| Watts (2007) | ★ | ★ | ★ |  |  |  | ★ |  | ★ | 5 | Poor |
| Seaman (2006) | ★ | ★ | ★ |  |  |  | ★ |  |  | 4 | Poor |

***C)*** ***Adapted NOS criteria for case-control study design***

For **case control studies**: a star (★) is achieved in each domain if following criteria are met:

#1: Is delirium definition adequate? (yes, with independent validation)

#2: Representative cases of patients with delirium (consecutive or obviously representative)

#3: Selection of comparative controls without delirium

#4: Definition of controls (no history of delirium)

#5: The study controls for age

#6: The study controls for severity of illness (e.g. APACHE or SOFA score) and neurological status (e.g. sedation and coma)

#7: Ascertainment of biomarker status (medical records or laboratory tests)

#8: Identical method of ascertainment for cases and controls? (yes)

#9: Identical non-response rate for both cases and controls? (yes)

| **CASE CONTROL STUDIES** | | | | | | | | | | | |
| --- | --- | --- | --- | --- | --- | --- | --- | --- | --- | --- | --- |
| **Author** | **Selection** | | | | **Comparability** | | **Exposure** | | | **Total score** | **Overall quality**  *(converted to AHRQ standards)* |
|  | *Adequate case definition* | *Representative cases* | *Comparative controls* | *Definition of controls* | *Study controls for age* | *Study controls for severity of illness and neurological status* | *Ascertainment of biomarker status* | *Same method of ascertainment* | *Same non-response rate* |  |  |
| ***First author (year)*** | ***#1*** | ***#2*** | ***#3*** | ***#4*** | ***#5*** | ***#6*** | ***#7*** | ***#8*** | ***#9*** |  |  |
| Wanderlind (2020) | ★ | ★ | ★ | ★ | ★ |  | ★ | ★ |  | 7 | Good |
| Voils (2020) | ★ |  | ★ | ★ | ★ |  | ★ | ★ |  | 6 | Good |
| Ozkul (2019) | ★ |  | ★ | ★ | ★ |  | ★ | ★ |  | 6 | Good |
| Grandi (2011) | ★ | ★ | ★ | ★ | ★ | ★ | ★ | ★ | ★ | 9 | Good |
