# Supplementary material for: Blood-based biomarkers and delirium in critically ill adults in the ICU: a systematic review
Source: Intensive Care Med Exp. 2026 Apr 20;14:48. doi: 10.1186/s40635-026-00894-5 (PMC13096256; doi:10.1186/s40635-026-00894-5)
Supplement: Supplementary file 2 — Supplementary material 2. [file 40635_2026_894_MOESM2_ESM.docx]

**Supplementary Figures A–H.**

Graphical overview of the direction and adjustment status of associations between blood-based biomarkers and delirium outcomes across included studies. The figures illustrate the distribution, directionality, and replication of findings across biomarker categories.

**Figure A:** Haematological markers


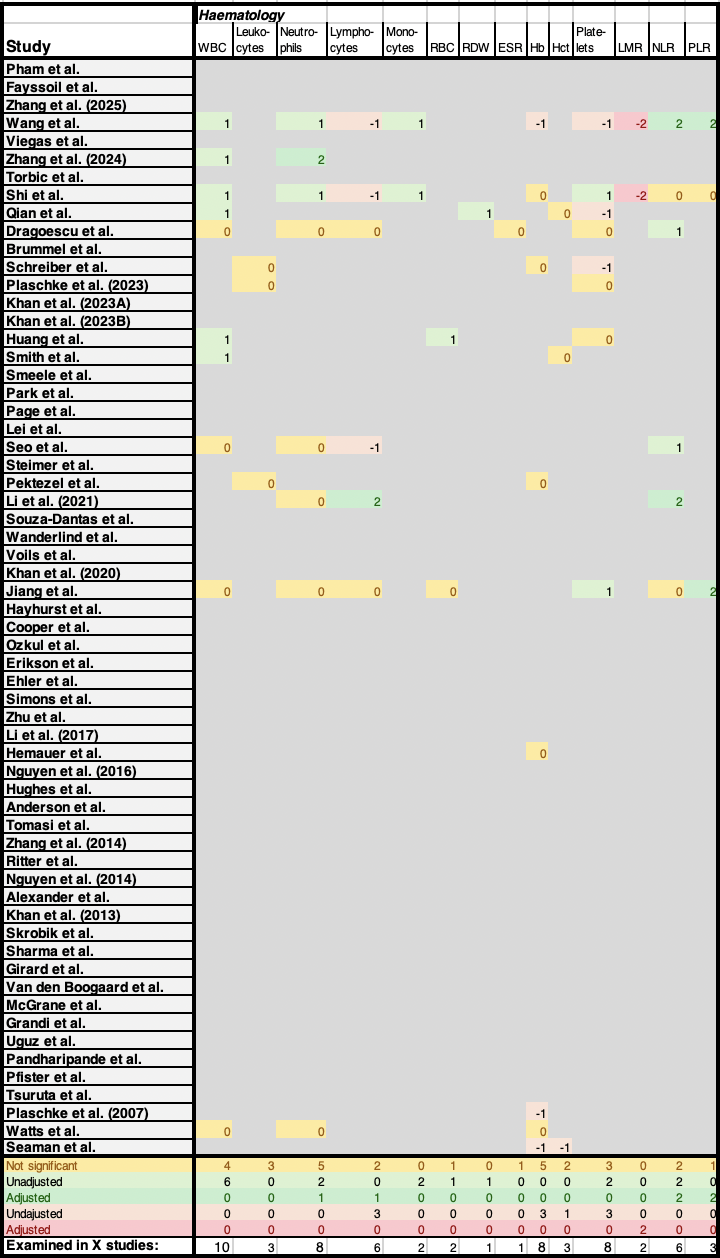


*Biomarker abbreviations: see Appendix B.*

Each row represents an individual study and each column a specific biomarker within the respective category. Associations are coded as follows: −2 = negative association in adjusted analysis; −1 = negative association in unadjusted analysis; 0 = non-significant; 1 = positive association in unadjusted analysis; 2 = positive association in adjusted analysis.

**Figure B:** Coagulation and endothelial dysfunction


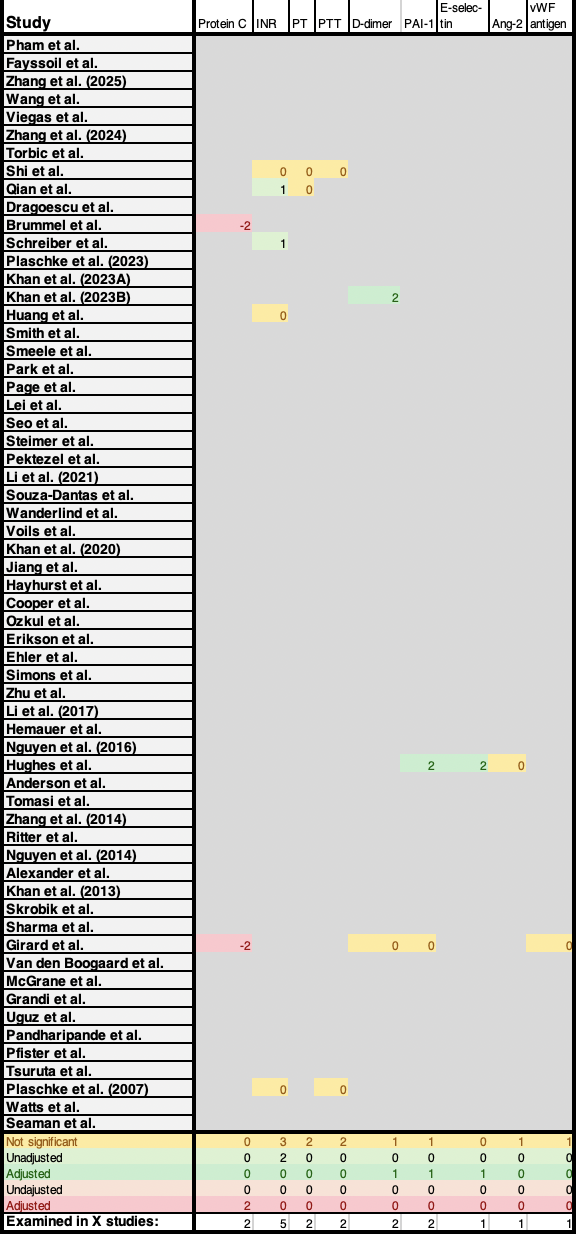


*Biomarker abbreviations: see Appendix B.*

Each row represents an individual study and each column a specific biomarker within the respective category. Associations are coded as follows: −2 = negative association in adjusted analysis; −1 = negative association in unadjusted analysis; 0 = non-significant; 1 = positive association in unadjusted analysis; 2 = positive association in adjusted analysis.

**Figure C:** Inflammatory, infectious and immunological markers


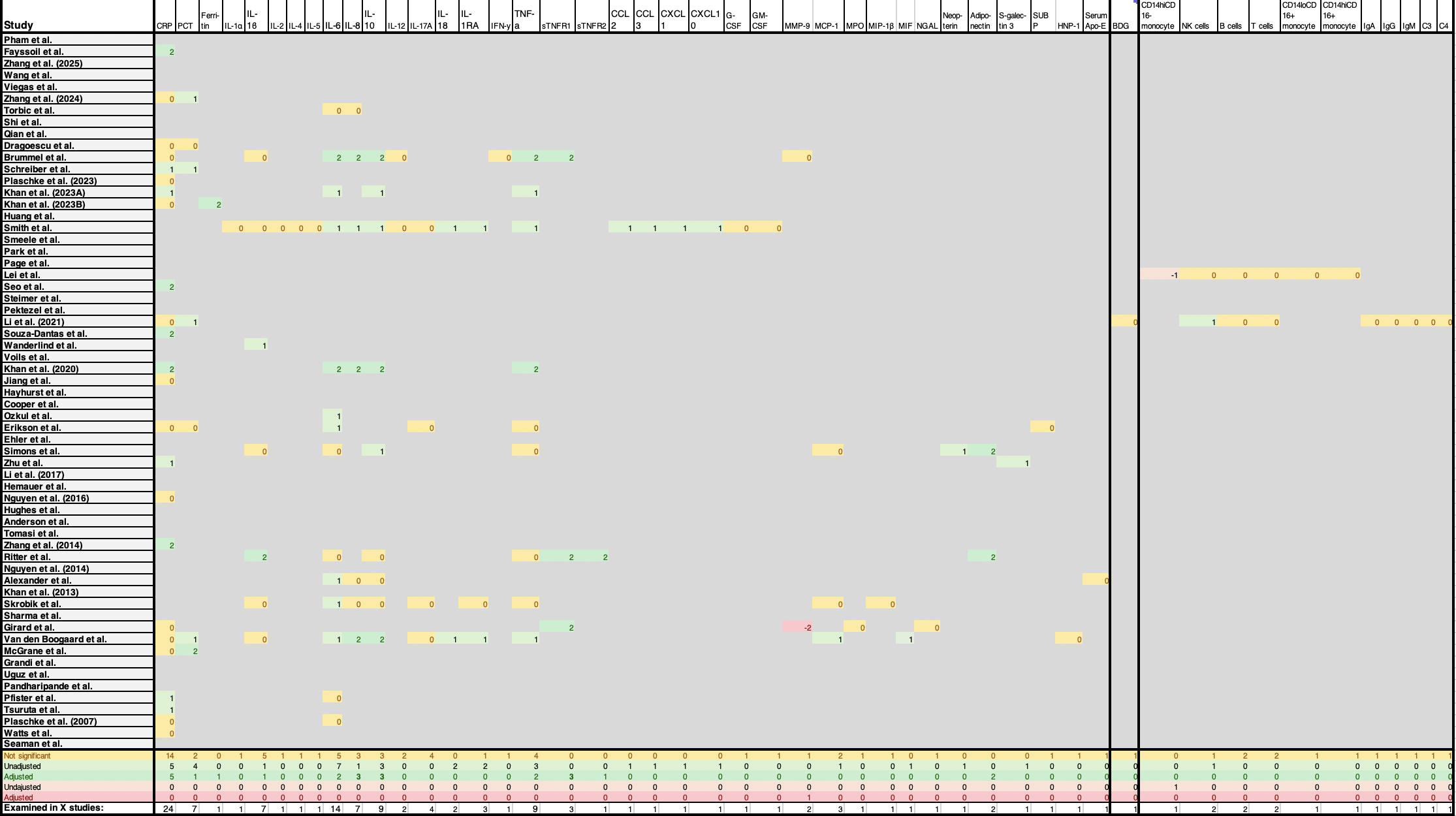


*Biomarker abbreviations: see Appendix B.*

Each row represents an individual study and each column a specific biomarker within the respective category. Associations are coded as follows: −2 = negative association in adjusted analysis; −1 = negative association in unadjusted analysis; 0 = non-significant; 1 = positive association in unadjusted analysis; 2 = positive association in adjusted analysis

**Figure D**: Neurobiological markers


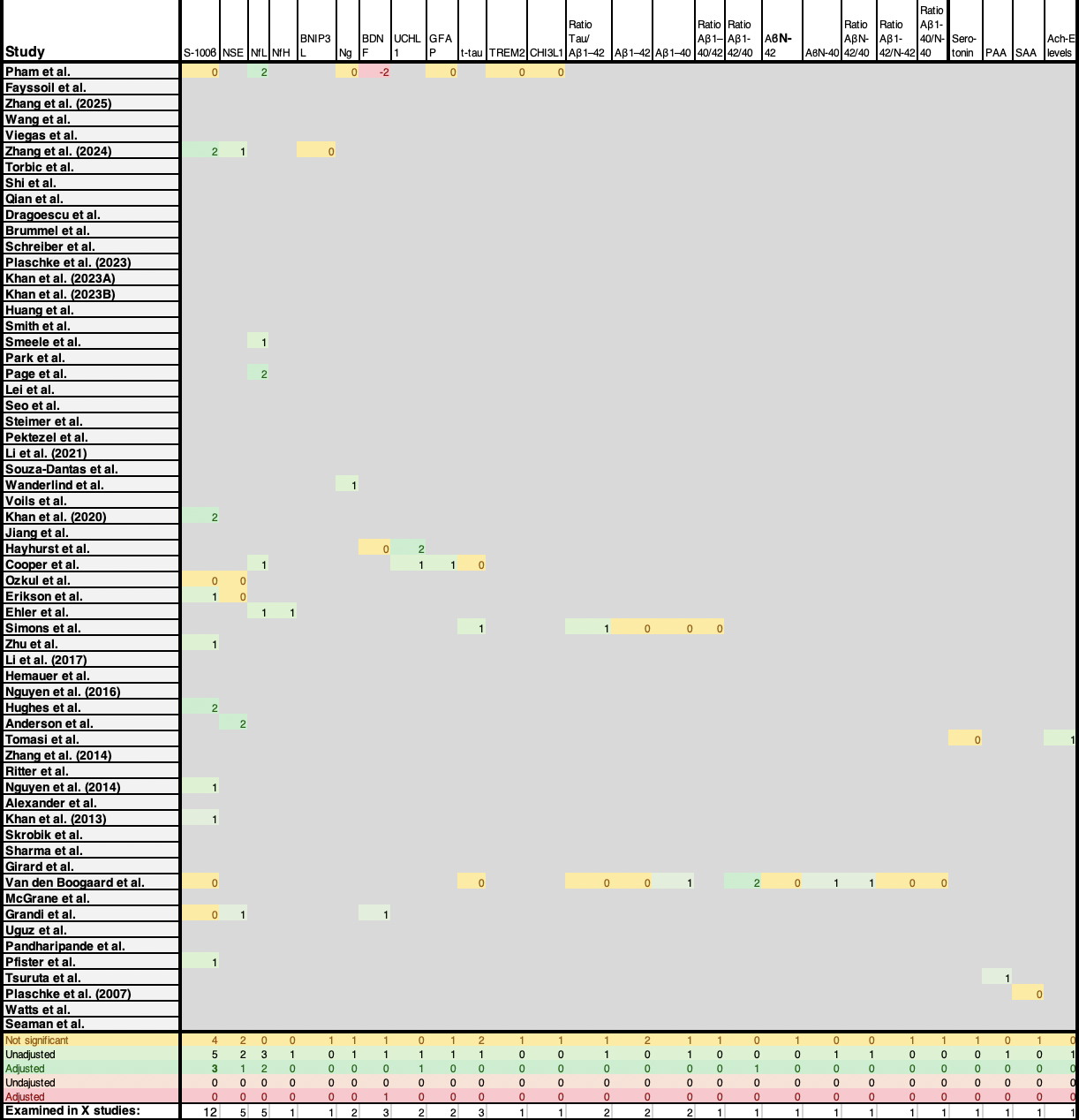


*Biomarker abbreviations: see Appendix B.*

Each row represents an individual study and each column a specific biomarker within the respective category. Associations are coded as follows: −2 = negative association in adjusted analysis; −1 = negative association in unadjusted analysis; 0 = non-significant; 1 = positive association in unadjusted analysis; 2 = positive association in adjusted analysis.

**Figure E:** Organ injury markers


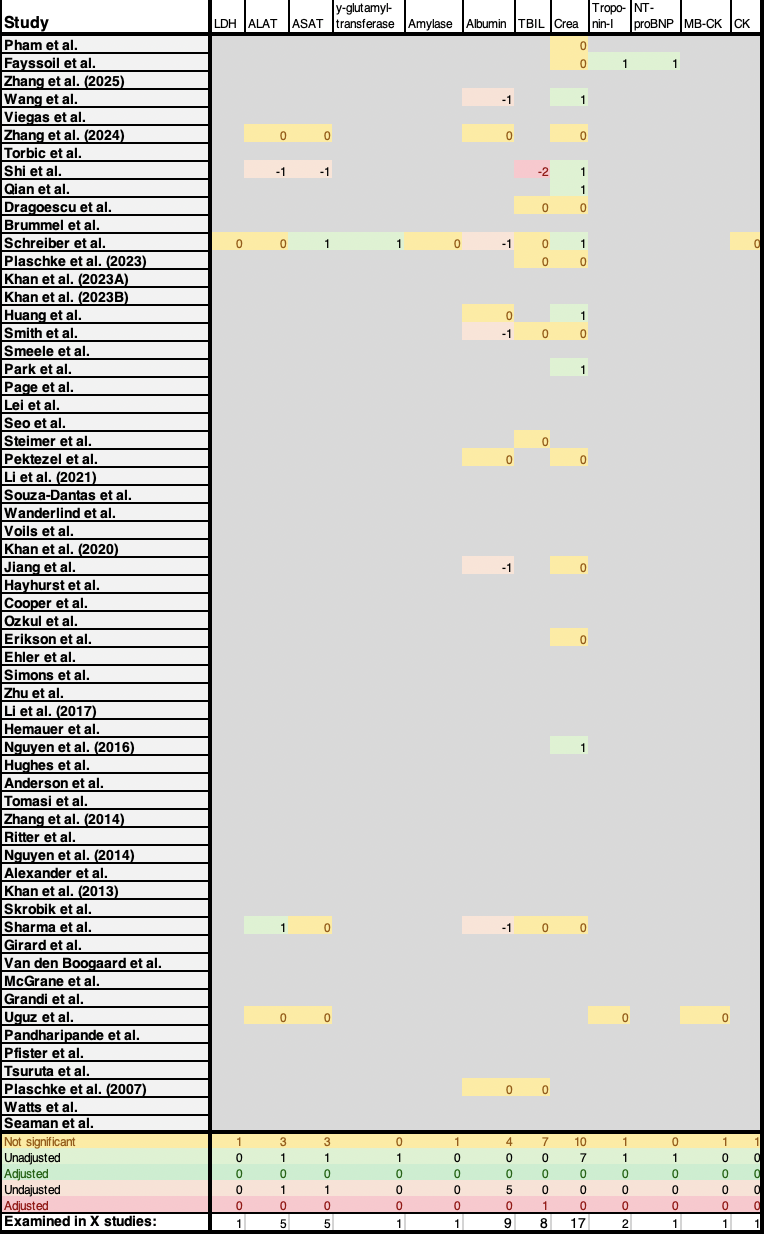


*Biomarker abbreviations: see Appendix B.*

Each row represents an individual study and each column a specific biomarker within the respective category. Associations are coded as follows: −2 = negative association in adjusted analysis; −1 = negative association in unadjusted analysis; 0 = non-significant; 1 = positive association in unadjusted analysis; 2 = positive association in adjusted analysis.

**Figure F:** Metabolic and endocrine function


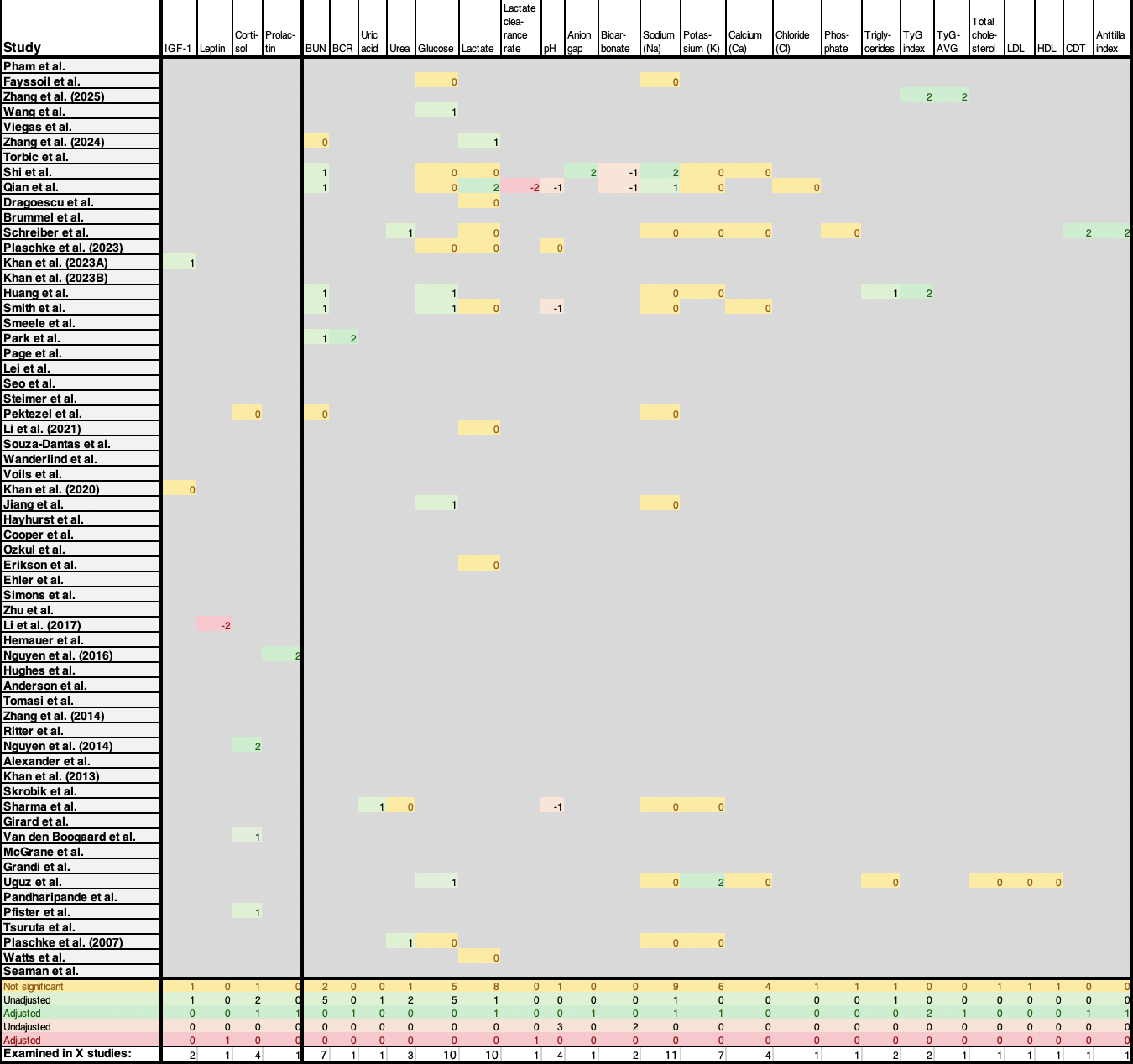


*Biomarker abbreviations: see Appendix B.*

Each row represents an individual study and each column a specific biomarker within the respective category. Associations are coded as follows: −2 = negative association in adjusted analysis; −1 = negative association in unadjusted analysis; 0 = non-significant; 1 = positive association in unadjusted analysis; 2 = positive association in adjusted analysis.

**Figure G:** Amino acid metabolism


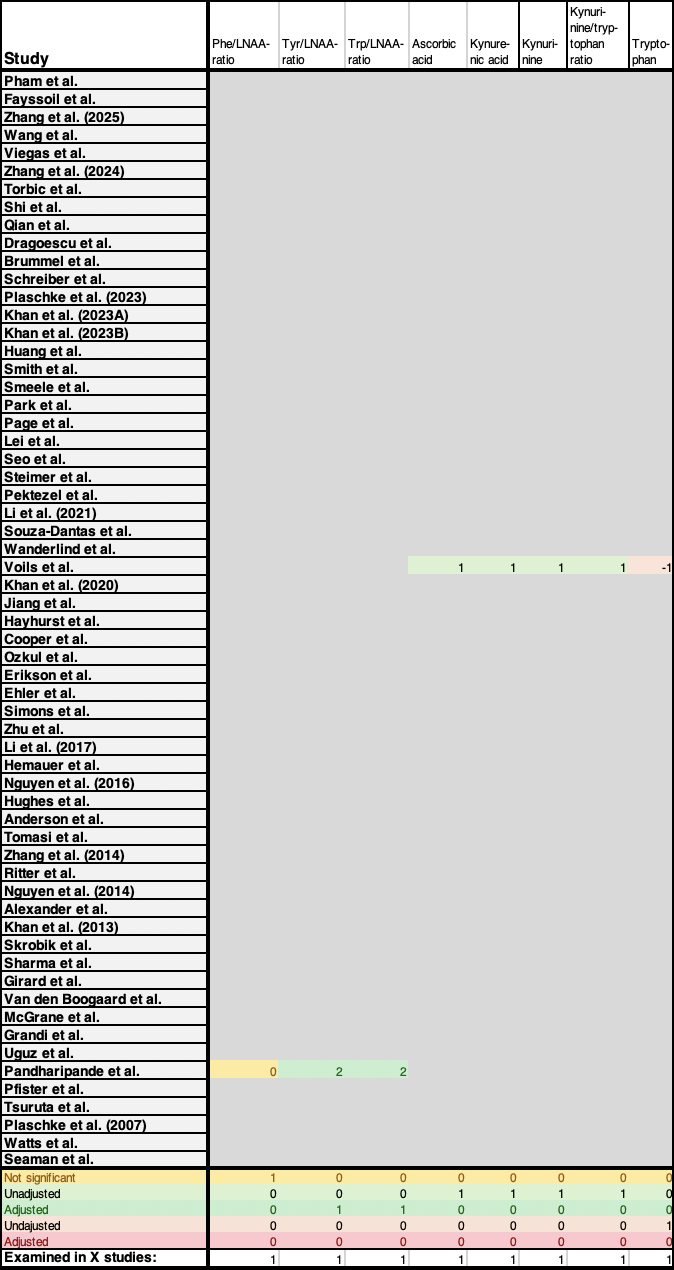


*Biomarker abbreviations: see Appendix B.*

Each row represents an individual study and each column a specific biomarker within the respective category. Associations are coded as follows: −2 = negative association in adjusted analysis; −1 = negative association in unadjusted analysis; 0 = non-significant; 1 = positive association in unadjusted analysis; 2 = positive association in adjusted analysis.

**Figure H:** Omics based exploratory markers


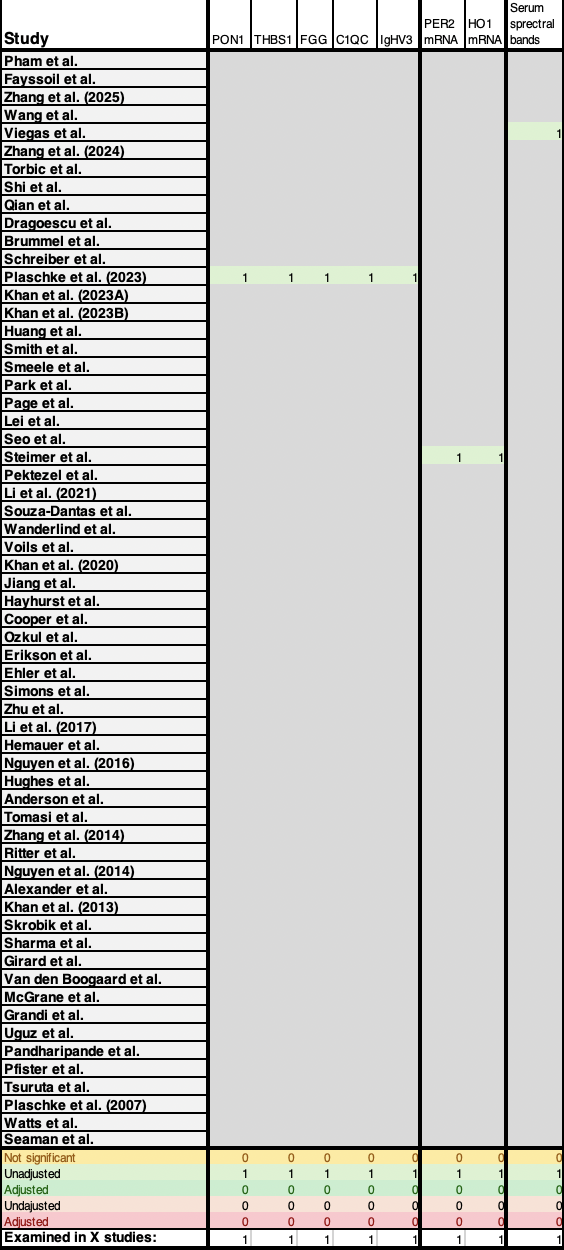


*Biomarker abbreviations: see Appendix B.*

Each row represents an individual study and each column a specific biomarker within the respective category. Associations are coded as follows: −2 = negative association in adjusted analysis; −1 = negative association in unadjusted analysis; 0 = non-significant; 1 = positive association in unadjusted analysis; 2 = positive association in adjusted analysis.
